# Bioelectrochemical biosensors for water quality assessment and wastewater monitoring

**DOI:** 10.1515/biol-2022-0933

**Published:** 2024-08-23

**Authors:** Anagha Bindu, Sudipa Bhadra, Soubhagya Nayak, Rizwan Khan, Ashish A. Prabhu, Surajbhan Sevda

**Affiliations:** Department of Biotechnology, National Institute of Technology Warangal, Warangal 506004, Telangana, India

**Keywords:** bioelectrochemical biosensors, wastewater monitoring, organic pollutant detection, biosensor design, pollutant detection

## Abstract

Bioelectrochemical biosensors offer a promising approach for real-time monitoring of industrial bioprocesses. Many bioelectrochemical biosensors do not require additional labelling reagents for target molecules. This simplifies the monitoring process, reduces costs, and minimizes potential contamination risks. Advancements in materials science and microfabrication technologies are paving the way for smaller, more portable bioelectrochemical biosensors. This opens doors for integration into existing bioprocessing equipment and facilitates on-site, real-time monitoring capabilities. Biosensors can be designed to detect specific heavy metals such as lead, mercury, or chromium in wastewater. Early detection allows for the implementation of appropriate removal techniques before they reach the environment. Despite these challenges, bioelectrochemical biosensors offer a significant leap forward in wastewater monitoring. As research continues to improve their robustness, selectivity, and cost-effectiveness, they have the potential to become a cornerstone of efficient and sustainable wastewater treatment practices.

## Graphical abstract

**Figure:**
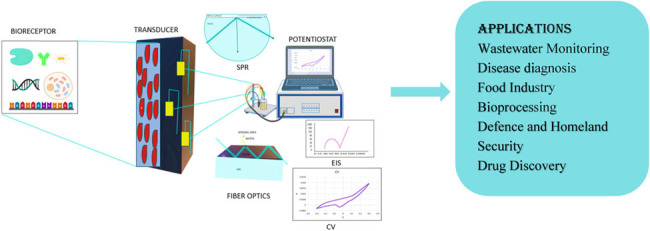

## 1 Introduction

In this modern era, certain areas of the world are still facing water scarcity, and conventional methods of treating wastewater have proven to be ineffective in treating issues such as biotoxicity, biochemical oxygen demand (BOD), and chemical oxygen demand (COD). Bioelectrochemical system (BES) is a recently researched remedy for focused and sustainable way of handling with real-time monitoring of wastewater [1]. BES is mainly focused on the potential of the interaction between electrode surfaces and microorganisms. The most used BESs consist of microbial fuel cells (MFCs), microbial electrolysis cells, microbial desalination cells, microbial solar cells, etc. These engineered systems enable living microorganisms to interact with electrodes, assisting various reactions that can be utilized for generating electric current, hydrogen production, desalination of water, and chemical production, respectively [2].

Since the MFCs did not specify more details on the electrodes where oxidation and reduction reactions are taking place, it was vital to explore further alternative methods. The development of biosensors seeks to improve the detection of biotoxicity and sensitivity. A biosensor is a bioanalytical instrument consisting of a bioreceptor to recognize and bind to the target analyte in order to produce a signal. Additionally, it contains a transducer to amplify and convert this signal to a desired form [1], and a signal processing unit that takes the output of the transducer to provide thorough information in terms of physical parameters and visualizing the information [3].

In recent years, biosensor has gained interest in various fields such as medical, industrial, agricultural, food safety, and environmental monitoring [2]. MFC as a biosensor works by forming a biofilm on the surface of electrode as a bioreceptor, proton exchange membrane (PEM), and anode electrode as transducer. The electrons produced from the biofilm travel to the anode electrode by a process called extracellular electron transfer. These electrons while travelling to the cathode electrode through the external circuit produce electric current, i.e., the transducer here converts the chemical energy into electrical energy and can be monitored in a visual interface. Biosensor due to its less maintenance and small size can also be used for remote areas [3].

Due to the improper discharge of industrial waste and household waste, water contaminations are increasing day by day because of which different types of waterborne diseases are also increasing and becoming life threatening [4]. These diseases are due to irregular monitoring of water quality, improper discharge of food materials, and lack of knowledge. Therefore, in order to decrease the mortality rate due to waterborne diseases, the real-time monitoring of water is important and that is only possible through biosensor [5]. The most known foodborne diseases are caused by *E. coli* species [6]. It is the indicator of coliform and fecal contamination in aquatic water [7,8,9]. Vu et al. developed an electrochemical biosensor by using carbon screen printed electrodes (SPEs) for the detection of *E. coli* O157 without label [10]. They used anti-*E. coli* O157 antibodies as the biological element and modified SPE with gold nanoparticles (AuNPs) act as the transducer. Electrochemical measurements of the developed biosensor were carried out by cyclic voltammetry (CV) and electrochemical impedance spectroscopy (EIS), which also detected *E. coli* O157 in the range of 106 CFU/mL [10]. Rochelet et al. developed a disposable screen-printed carbon sensor for the detection of β-d-glucuronidase (GLUase) activity of *E. coli* and monitored using CV. The data show that the obtained anodic current was linear to the amount of GLUase and detected 10 ng/mg of GLUase [11]. The purpose of this review is to draw the attention to the latest and most researched technology of BES-based biosensor, to know the main application field associated with it, and to know the major challenges and scalability of bioelectrochemical biosensor in near future.

## 2 Principles of bioelectrochemical biosensors

Electricity is the main energy source, without it nothing is possible in this modern world. There are different sources to generate electricity such as hydroelectric power, solar panels, wind mills, etc., but these methods are expensive and require large space to operate it. Alessandro Volta in 1793 was the first to produce battery by inserting wet paper in between silver and zinc metals and showed electricity can be generated [12]. The journey of electrochemical process started in eighteenth century [13], which then led to bioelectrochemical process. In 1911, Potter produced electricity from *Saccharomyces cerevisiae* and a maximum of 0.3–0.5 V was recorded [14]. This led to the start of using different types of MFCs and microbes in order to produce maximum electricity. The power density of MFC has increased from 0.001–0.01 mW/m² in 1992 [15] to 3,600 mW/m² in 2003 [16] to 8486.42 mW/m² in 2021 [17].

**Figure 1:**
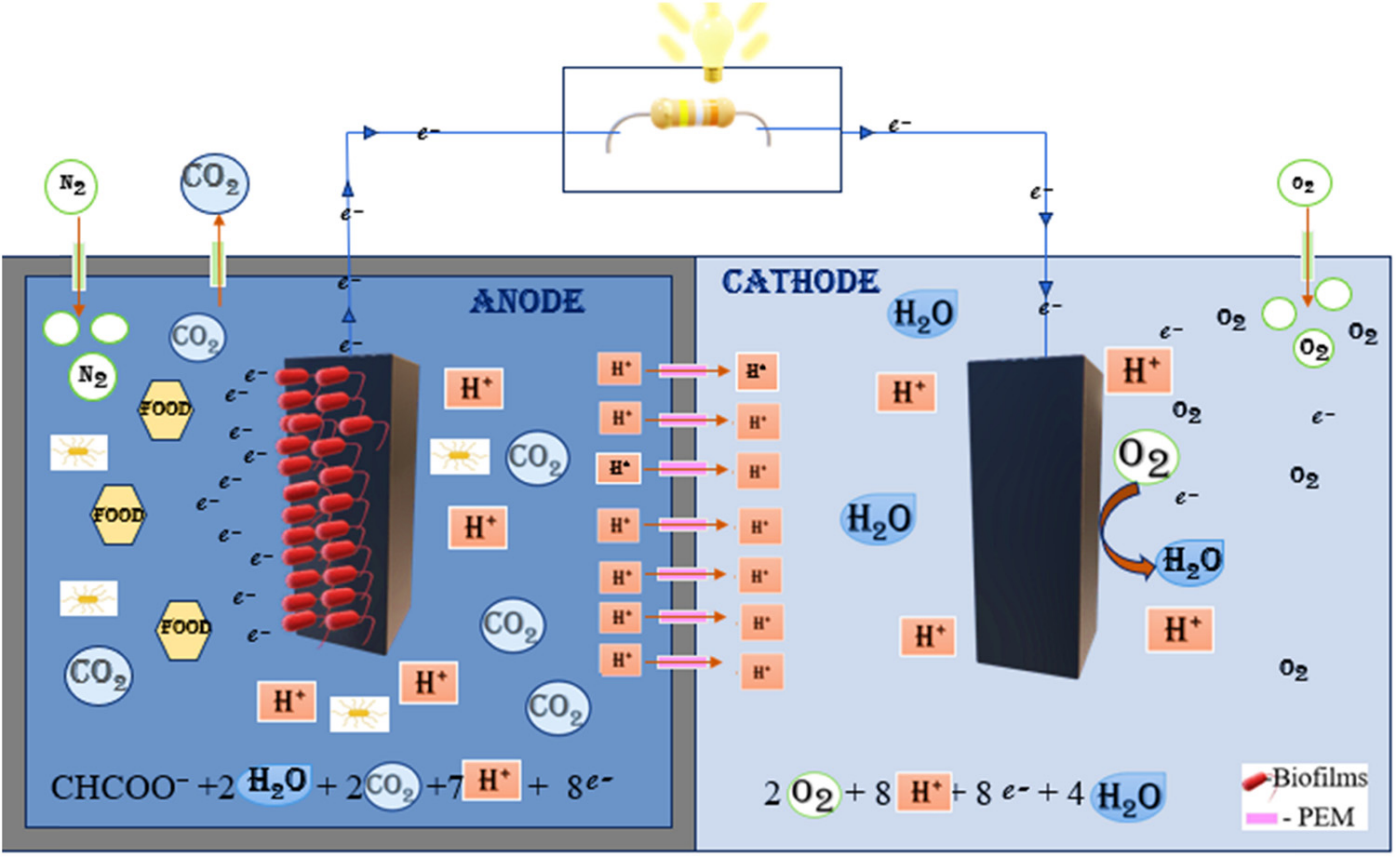
The basic working principle of BES, showing anode and cathode chambers with electrodes and biofilms attached.

BES uses electroanalytical techniques to measure the current by converting the chemical energy stored in the organic matter with the biodegradable activity of microorganism (Figure 1).

The main aim of BES is to make electrochemical connection between microorganism and the specific electrode. The most studied and researched BES for electricity production is MFC. According to Murugesu et al., the research and demand for MFC have increased over the last 20 years and China is the leading country with almost 4,000 published articles after US, India, and South Korea [18]. A dual chamber MFC consists of an anode, a cathode, and a PEM. The microorganisms capable of biodegrading the organic matter and producing electrical energy are called electrogens.

Electrogens are electrochemically active bacteria which oxidize the organic matter anaerobically to release electrons, CO₂, and protons in anodic or anaerobic chamber. The travelling of electrons to cathode through the external circuit creates bioelectricity. The reactions taking place in anode and cathode chambers of an MFC are as follows:

Anaerobic chamber

\[{\text{CHCOO}}^{-}\hspace{.5em}\text{+}\hspace{.5em}{\text{2H}}_{\text{2}}\text{O}\hspace{.5em}\text{+}\hspace{.5em}{\text{2CO}}_{\text{2}}\hspace{.5em}\text{+}\hspace{.5em}{\text{7H}}^{\text{+}}\hspace{.5em}\text{+}\hspace{.5em}{\text{8e}}^{-}.]\]

Aerobic chamber

\[2{\text{O}}_{2}+8{\text{H}}^{+}+8{\text{e}}^{-}+4{\text{H}}_{2}\text{O}.]\]

**Figure 2:**
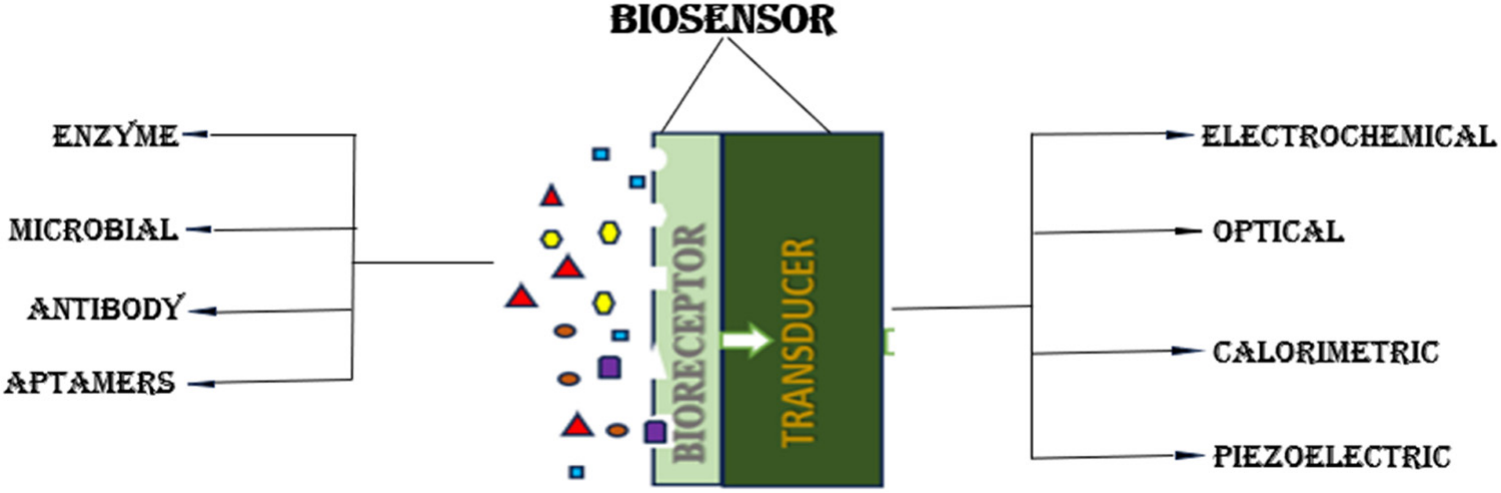
The classification of biosensor based on bioreceptor and transducer parts.

The protons released during the oxidation reaction reaches the cathode by PEM. In cathode or aerobic chamber, which is in contact with air, the reduction of oxygen takes place and produces water as byproduct by combining with proton and electron [19]. BES biosensor has been developed for its fast, efficient, and cost friendly behavior. The key components of a biosensor are biological recognition element, an integrated transducer, and a signal processing unit. After adding the wastewater, the biofilm starts to form on the electrodes, electrogens attached to the electrode will act as the bioreceptor that will detect and bind to the specific target analyte. The anode acts as a transducer, which is a converter that converts the biochemical signal recognized to electrical signal and amplifies it. Then, the signal is delivered to a data processor where the output is converted to a physical parameter and visually analyzed. Biosensors can be categorized based on various types of transducers and bioreceptors used, and customized for different types of applications in various fields (Figure 2).

Based on transducer part, biosensor can be electrochemical, optical, calorimetric, and so on, while on the bioreceptor part, biosensor can be enzyme-based, microbe-based, antibody-based, aptamer (Apt)-based, and even the latest technology uses nanomaterial-based biosensors. Biosensors are classified according to different bio-transduction and biological signaling methods operated for the recognition of elements. Transducer is a converter system that detects signals from the interactions of biological receptor with the analyte and transforms biochemical signal into measurable electrical output [20]. Table 1 gives a summary of contaminants found in wastewater and the biosensor used to detect these contaminants.

**Table 1:** Contaminants found in wastewater and the type of biosensor used to detect the contaminants

Contaminants found in wastewater	Analytes	Biosensors	References
Heavy metals	CdCl_2_	1D phononic crystal cavity sensor	[21]
	2,4-dinitrotoluene	Whole-cell biosensors	[22]
	Pb^2+^, Cd^2+^, and Cr^3+^	Hybrid nanoparticle/DNA zyme electrochemical biosensor	[23]
	Toxic heavy metal ions	Colorimetric aptasensor based on AuNPs	[24]
Dyes	Azo dyes	Manganese peroxidase based voltammetric biosensor	[25]
	Rhodamine detection	Surface-enhanced Raman scattering sensors	[26]
	Indigo carmine dye	Electrochemical biosensor	[27]
Pesticides	Atrazine	Opto-electrochemical biosensor	[28]
Pharmaceutical compounds	Ampicillin	Electrochemical biosensor	[29]
	Ofloxacin	Photoelectrochemical	[30]

Based on recent advancements and functionalities, there are a number of biotransduction methods available, they can be further grouped into labelled and label-free types, in which label-free biotransduction methods are most widely developing recently.

## 3 Electrochemical biosensor

This is an important class of biosensor in which the electrode is employed as a biotransduction element [31]. Biotransducers due to their portability, high sensitivity, and simplicity have a lot of advantages. Electrochemical biosensor is a self-sufficient integrated system, which uses a biological recognition component coupled to an electrochemical transduction element to measure quantitative or semiquantitative analytical information [32]. The monitoring of electrochemical change can be done by (i) change in potential difference between the electrodes (potentiometric), (ii) change in measuring the impedance value (impedimetry), (iii) change in measuring the electrolytic conductivity (conductometric), and (iv) change in measuring the current at a given voltage (amperometric) [2,33]. Electrochemical biosensors are analytical tools that combine biological recognition elements with electrochemical transducers. Table 2 summarizes their key components and applications.

**Table 2:** Components and application of electrochemical biosensors

Components	Description
Biological recognition element (bioreceptor)	Molecule (enzyme, antibody, etc.) that specifically binds to the target analyte
Electrode	Conductive surface where the electron transfer between the bioreceptor and the transducer occurs. Common materials include gold, platinum, and carbon-based materials (nanotubes, graphene)
Transducer	Converts the biorecognition event into a measurable electrical signal (current, voltage, impedance)
Applications	Medical diagnostics (glucose monitoring, detection of pathogens) – environmental monitoring (pollutant detection) – food safety testing – biosecurity applications

### 3.1 Potentiometric biosensor

The potential difference between electrodes is measured by potentiometric biosensor. Here the bioreceptor converts the recognition signal into potential signal. That is, fluctuations in analyte concentration can create a change in potential, and this potential change is recognized for analysis. It was first reported for the detection of urea in 1969 using a 2-electrode setup [34,35]. It can be 2-electrode or 3-electrode or 4-electrode setup. Most commonly used one is 3-electrode setup, consisting of working electrode (whose potential is unknown and to be measured), reference electrode (whose potential is known and used to measure the working electrode), and counter electrode (which completes the electronic circuit). The potentiometric transduction works on the principle that when zero or no current is flowing, the potential difference between the working and reference electrode is measured. Also, it will give the information about the oxidation and reduction of ions in electrochemical reaction [36]. This measurement is calculated by the Nernst equation which tells the relationship between concentration and the potential difference [31]. For example, with an ammonium-targeted electrode, the suggested potentiometric flow biosensor by Zhang et al. effectively measures the diminution of *Nitrosomonas europaea*’s ammonia-oxidizing activity to determine water pollution. It is a useful instrument for identifying harmful materials and monitoring the quality of water because of its great sensitivity and fast reaction time [37].

### 3.2 Conductometric biosensor

The conductometric transduction measures the conductivity of the electrolyte with respect to the concentration of ions, in other words, it measures the current that can be conducted by an electrolyte solution. When kept between two electrodes in electrolyte solution, the electric current will pass through it based on the release and uptake of ions. The conductance measurement is fast and highly sensitive but the sensitivity of the conductometric biosensor is still questioned and research to overcome this is still ongoing. Conductometric biosensor can be used to analyze arginine, urea, and glucose. Also, it has many advantages such as it can be operated at low amplitude, it can used in varying light environment, it can be incorporated with other electronic devices, and also, it can be operated even without reference electrode [33,38].

### 3.3 Voltammetric transduction biosensor

Voltammetric transduction provides current voltage relationship at specific electrode. Voltammetry measures the potential difference between working electrodes with respect to the reference electrode in order to measure the subsequent current [39]. The origin of voltammetry was from polarography by the Czech chemist Jaroslav Heyrovsky in 1922 [40]. It uses three electrodes, the working electrode (mercury, glassy carbon, graphite, gold), reference electrode (calomel electrode, silver/silver chloride electrode), and auxiliary electrode (thin platinum wire, graphite, and gold) to give relationship between potential, current, and time. The principle is that in a given period of time, the resulting electrical current passing through the bioelectrochemical electrode is captured, when potential is applied to the working electrode. The curve between applied potential vs current is called voltammogram, which is highly sensitive, selective, and can be operated in varying pH and temperature conditions.

CV is the most used voltammetric method for measuring the current by applying potential. It gives information about the redox behavior, diffusion (flow of electrons) of working electrode as a function of given potential. In CV, voltage scan is linearly varied, the scan will start from one potential, continues in reverse potential, and comes back to the start potential to give the electrical current values at different voltages [41]. Scan rate is the speed of voltage variation occurring per second. It is required to understand the intermediate reaction happening, stability of products, diffusion of electrons, and for the study of redox process.

Linear sweep voltammetry is a voltammetric method where current is measured by giving voltage linearly. It is a half cycle of CV, where the scan will only flow from the lower potential to upper potential (forward direction) with known scan rate to give the subsequent current values. It is used to know whether oxidation or reduction is happening in the electrode, redox properties of analyte, electron transfer rate, and concentration of analyte. The peak indicates oxidation or reduction occurred at that potential range. Differential pulse voltammetry (DPV) is a type of pulse voltammetry technique, in which a series of short voltage pulses (10–100 mV) are applied linearly over time to the working electrode. The base potential (initial potential) is equally increased with respect to each pulse. The current is differentially measured before and after each voltage step. For instance, the generation of a very sensitive electrochemical DNA biosensor can quickly identify Vibrio cholerae genetic material in environmental materials, such as vegetables and water. Method of recognition used a sandwich DNA hybridization method where the complementary DNA of *V. cholerae* was flanked by a reporter probe and a capture probe. The hybridization was measured using DPV in spite of an anthraquinone redox tag. The bacterial counts acquired using conventional microbiological techniques showed a strong correlation with the concentrations found by the genosensor [42]. The sensitivity of DPV is more due to small capacitive current as short duration pulses are applied. The main advantage of DPV over CV is that it is used to find out similar oxidation potentials among different analytes.

### 3.4 Impedemetric biosensor

EIS-based transduction measures the impedance by applying very low sinusoidally varying voltage over a frequency range. Schulze and Lorenz in 1975 were the first to use EIS for knowing the resistance and capability of a material [33]. Impedance is the opposition to current flow similar to resistance in an electrical circuit. The frequency is varied from mHz to MHz, while sweeping through these frequencies, EIS provides information about the electrode processes at various time scales. In simple words, it is the perturbation from equilibrium or steady state of electrochemical system by applying sinusoidal ac voltage [43].

Nyquist plot and Bode plot are the two standard curve representations obtained in an EIS to describe the kinetic and mechanistic details of electrochemical system. Nyquist plot gives relationship between charge transfer resistance and solution resistance by plotting the real part of impedance in *y* axis and the imaginary part is displayed in *x* axis. It consists of two parts, semicircle and Warburg element. Semicircle portion of the plot gives data about the charge transfer resistance and the diagonal line represents the Warburg element which gives an idea about the movement of ion in solution or at the interface. Bode plot which is a superset of phase plot (displays phase angle over a range of frequencies) and magnitude plot (displays amplitude of impedance over a range of frequencies) gives information about the frequency-dependent behavior of the system. EIS phenomenon is mainly useful in deducing the impedance method (to know the resistance, capacitance, inductance over a range of frequencies), active species diffusion, double layer capacitance, and electrical charge transfer process at electrode interphase. The major drawback is that it will give false positive detections and hence it is less used compared to potentiometric.

## 4 Optical biosensor

Optical biosensor has become more advance in the past few decades due to its vast applications in medical diagnosis, research purposes, environmental monitoring, and food safety [44,45,46]. It is a label-free, real-time transduction method capable of detecting various types of target materials [47]. This type of transduction uses many types of spectroscopies such as absorption [48], fluorescence [49], phosphorescence [50], surface-enhanced Raman scattering [51], refraction, and dispersion [52] spectroscopy in order to be employed as a biosensor.

Optical means light, i.e., the output signal is light. It converts biological analyte using optical signal due to its high transduction efficiency. Optical biosensors utilize light interaction with a biorecognition element to detect the presence or quantity of a target analyte. Table 3 summarizes their key features.

**Table 3:** Components and application of optical biosensors

Components	Description
Light source	Provides light to interact with the biorecognition element. Common sources include lasers, LEDs, or broadband light sources
Biorecognition element	Similar to other biosensors utilizes biomolecules (enzymes, antibodies, etc.) for specific target binding [53]
Transducer	Converts the interaction of light with the biorecognition element into a measurable optical signal. This can involve changes in intensity, wavelength, polarization, or propagation characteristics of light [54]
Detection system	Detects and measures the modified optical signal [53]
Applications	Wide range: medical diagnostics (immunosensors, DNA analysis), environmental monitoring (pollution detection), food safety testing, drug discovery [53]
Advantages	Label-free detection (in some cases). High sensitivity and specificity. Real-time monitoring capabilities
Disadvantages	Can be complex and expensive to develop. May require sophisticated instrumentation for detection

The most common example for optical biosensor is glucometer for the detection of blood glucose level. It is available in two modes: label-free and labelled modes. Label-free mode detects direct signal from the interaction of bioanalyte and biotransducer, whereas labelled mode requires labels like fluorescent tags to detect the optical signal. Optical transduction occurs when sensor surface shows changes such as scattering, refraction, and reflection [55]. In labelled mode, the detection is limited to single molecule and can interfere with biomolecule functions which becomes major drawback because of which label-free optical biosensors are preferred [56].

### 4.1 Surface plasmon resonance (SPR) biosensor

Liedberg et al. in 1983 was the first to demonstrate SPR for explaining the boundary conditions and gas detection [57]. It uses electromagnetic waves to detect signal during the interaction of analyte and biological element. The principle behind its working is change in refractive index induces measurement of the reaction. That is, when the biorecognition element is immobilized on sensor surface and the target analyte binds to the biological element, there is a change in refractive index in the surface of sensor. This change can be detected as optical by sensing transduction signal [55]. In other words, it is a physical phenomenon which occurs at the surface of conductive material when excited by a polarized light of certain angle [58]. In order to measure the absorption of sample analyte, a spectrophotometer is used. SPR can be used to detect binding of small analytes, i.e., as small as 2 kDa [59]. SPR-based biosensor has a wide range of applications such as to find the short sequence of nucleic acids [60], to detect pathogens and diseases [44], drug discovery, environmental monitoring, and biomedical research [61].

Optical fibers/optrodes have garnered significant attention for biosensor development, especially in applications requiring lower detection limits. Biosensors based on optical fiber typically consist of key components: a light source, an immobilized bioreceptor element, an optical fiber transmitting light and serving as the substrate, and a detector (e.g., spectrophotometer) where the output light signals are measured. Hence, in fiber optic biosensor, a light source will traverse optical fibers housing immobilized bioreceptors, reaching a photon detector [62]. When the target analyte interacts with the bioreceptor on the fiber’s surface, a biochemical reaction occurs, leading to changes in optical properties. The binding of analytes and the subsequent introduction of a suitable labelling reagent induce a signal alteration at the detector. Hence, due to its major advantages, namely, portable high-performance sensors, high sensitivity, fast responses, high selectivity, and low detection limits, optical fibers are gaining higher amounts of research interests [63].

### 4.2 Calorimetric-based biosensor

The integration of immobilized enzymes into bioanalysis generated curiosity among several research teams to create affordable, straightforward calorimeters for regular use. This is particularly essential in clinical settings where there is a need to accurately determine metabolites such as glucose and urea [64]. The biosensing devices based on calorimetry is greatly influenced by heat generation and heat absorption occurring during biochemical reactions. At the starting, calorimetric-based transduction was utilized in enzyme biosensors, later it has found a wide variety of applications in cell and immunosensors [65]. The principle of calorimetry implies measuring the temperature changes resulting from the interaction between the biorecognition element and a compatible analyte. These temperature changes are related to the amount of reactants that are used or the products produced.

A new biosensing method has been developed by Hundeck in 1993 to detect and measure enantiomeric excesses (EEs) in aqueous and organic environments [66]. EE shows the addition of one enantiomer compared to its counterpart in a racemic mixture. Precise determination of EE holds significant importance throughout a variety of sectors including pharmaceuticals, agrochemicals, and fine chemicals. The biosensor discussed in the paper employs calorimetric transduction principles to quantify heat variations resulting from the interaction between chiral analytes and a particular biorecognition element, such as enzymes, antibodies, and Apts. The calorimetric biosensor developed employs bioreceptor elements, such as enzymes or antibodies, immobilized onto the surface of a sensor to specifically bind one enantiomer over the others. This binding interaction leads to the generation of a detectable heat signal, allowing for the correlation of this signal with the EE present in the sample. The sensitivity of calorimetric-based biosensor can be increased by combining with microfluidics. For instance, Leake and colleagues created a weight-oriented sorting method on a planar optofluidic device by using optical forces for micro- and sub-micron particles. The technique allows for high efficiency and high sensitivity, making it useful for many applications which requires low-level size detection such as food safety and environmental monitoring [67].

More recently, the calorimetric-based biosensor method has found many applications such as (i) food and water safety and quality control by identifying pollutants, pathogens, or other analytes [68]; (ii) with the use of these biosensors, biologists can better understand biological systems by investigating a range of biochemical processes, identifying biomolecules, and keeping an eye on cellular activity [69]; (iii) vital in medical diagnostics to identify pathogens, disease biomarkers, or genetic material, providing a quick and precise diagnosis of a variety of illnesses [70]; (iv) through analysis and characterization, these techniques offer insights into the characteristics, interactions, and potential uses of nanomaterials in a variety of fields, such as materials science and medicine [67]; and (v) to analyze the quality of the environment, find dangerous materials, and determine pollution levels, all of which help to safeguard public health and ecosystems [71].

### 4.3 Piezoelectric-based biosensor

A piezoelectric biosensor is an instrument that monitors alterations in a biological specimen’s weight, viscosity, or other physical characteristics using the piezoelectric effect. In other words, when a material produces an electrical current in reaction to mechanical stress such as variations in vibration or pressure, this phenomenon is known as the piezoelectric effect [72]. The connection between mass and resonance frequency is the fundamental idea behind piezoelectric biosensors. A piezoelectric substance, such as quartz [73] or lead zirconate titanate [74], is used as a sensing component in piezoelectric biosensors. Electrodes are usually put on both sides of a crystal sheet or thin layer that contains the substance. Applying a biological specimen to the sensor causes the piezoelectric material to connect with the sample, changing the material’s mechanical characteristics including mass, viscosity, and elasticity [31]. The mass of the piezoelectric crystal is changed by a biomolecule’s interaction with the sensor surface, which modifies the crystal’s resonant frequency.

Numerous publications have been made, most of which are intended for use in the medical field [75]. Other essential considerations have been made, in addition to the traditional utilization of the antibody–antigen interaction and the significant benefit of piezoelectric transduction, which are essentially a mass balance and do not require labelling or the employment of a secondary antibody. First, it appears that the phase of immobilization of antibodies is still the most researched in the creation of piezoelectric immunosensors. In fact, piezoelectric transducers are possibly a highly flexible and suitable instrument for researching immobilization techniques, which may subsequently be used to various systems for the creation of biosensors and bioassays [76].

**Figure 3:**
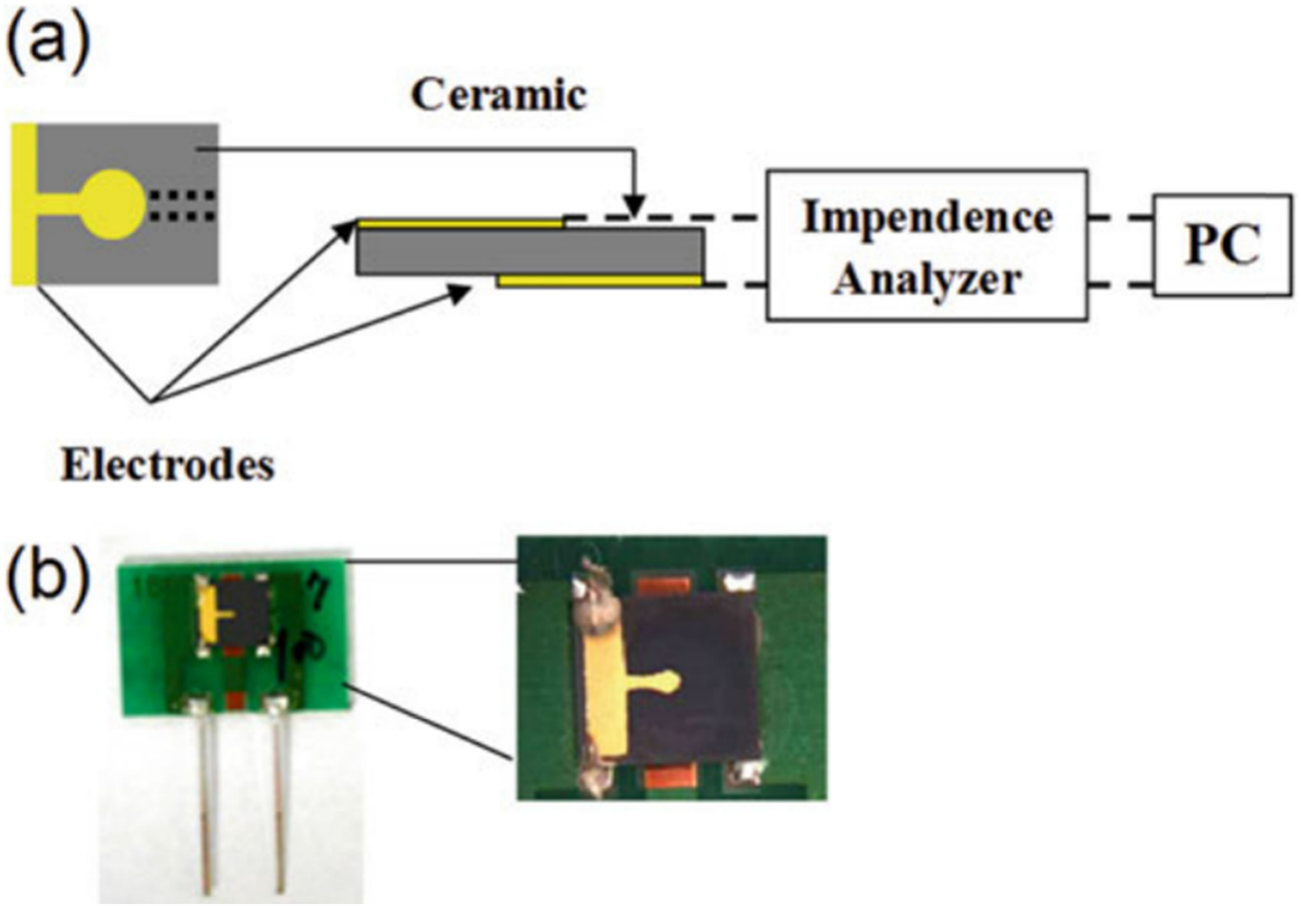
A PZT-based piezoelectric biosensor for the early detection of cancer markers. (a) Shows various layouts of the ceramic resonators and (b) a photograph of the ceramic resonator typically shows a small, disk-shaped, or rectangular component with electrodes attached (40 MHz ceramic resonator) taken from Su et al. [74].

With an emphasis on alpha-fetoprotein (AFP), Chen and Shi created a continuous flow immunoassay instrument utilizing a piezoelectric quartz crystal sensor for the precise molecular detection of tumor indicators. The instrument can detect AFP quantities ranging from 18.8 to 1,100 ng/mL, and the immunoreaction only takes 12 min. Samples of serum from a variety of medical sources, including expectant mothers, patients with lung and liver cancer, and individuals having liver cancer screening, were used to test the device. The analysis’s findings showed good accuracy and reliability, nearly matching those from radioimmunoassay. There was no interference with lung cancer markers, indicating the instrument’s excellent sensitivity for AFP [73]. Additionally, a novel biosensor was created for the direct identification of cancer markers, utilizing a piezoelectric polycrystalline lead zirconate titanate (PZT) ceramic resonator. One resonator was linked in parallel to act as the unit that senses and the other as the control component in the dual ceramic resonator scheme used in the layout shown in Figure 3. By monitoring variations in frequency, cancer indicators, namely, prostate specific antigen and AFP, were identified. Diagnostic standards for measurement were met by the biosensor, which showed excellent sensitivity (0.25 ng/mL), quick detection (within 30 min), and minimal sample quantities (1 μL). The outcomes of the investigation demonstrated that this piezoelectric biosensor based on ceramic resonators may be modified to suit different chemical interfaces and has the potential to be developed into biosensor arrays that can detect numerous analytes at once [74].

## 5 Biological elements in BES-based biosensors

Bioreceptors, or biological recognition elements, are what distinguishes a biosensor from a sensor. The biological element is the heart of a biosensor which plays an important role in the sensing of analyte part in the whole mechanism. These bioreceptors can be enzymes, antibodies, nucleic acids, or whole cells that specifically interact with the target analyte. They enable the biosensor to selectively detect and measure the presence of the analyte in a sample. If a bioreceptor is not able to recognize any analyte, then the whole biosensor is an ultimate failure. So, the core part of any biosensor is bioreceptor. Bioreceptor is a biomolecular part that requires biochemical mechanism for detection. Bioelectrochemical biosensors rely on biological recognition elements to achieve specific and sensitive detection of target analytes. Table 4 summarizes the most common biological elements used.

**Table 4:** Common biological elements used in BES-based biosensors

Biological element	Description	Example
Enzymes	Proteins that catalyze specific biochemical reactions. Often used because they convert the target analyte into a product that alters the local environment for electrochemical detection	Glucose oxidase (Gox; detects glucose by generating H^+^). Lactase (detects lactose by hydrolyzing it)
Antibodies (immunoglobulins)	Highly specific proteins that bind to a unique epitope (region) on a target molecule (antigen). Useful for detecting pathogens, toxins, or specific proteins	Anti-cancer antibodies (detect cancer markers). Anti-bacterial antibodies (identify specific bacterial strains)
Apts	Single-stranded nucleic acid molecules (DNA or RNA) engineered to fold into specific 3D structures that bind to target molecules with high affinity and selectivity	Apts for toxin detection. Apts for monitoring specific cell types
Whole cells/organelles	Living cells or organelles (e.g., mitochondria) can be integrated into biosensors to leverage their natural metabolic processes for analyte detection	MFCs for pollutant detection. Yeast cells for monitoring fermentation processes

The choice of biological element depends on the specific target analyte and desired detection mechanism.

Bioreceptors should be sensitive and selective as per to identify the specific target to overcome interaction with other materials in a solution. Based on different types of biosignalling methods utilized, the biosensor can be subclassed as enzyme, antigen/antibody, whole cell/cell (bacteria/phages/virus), nucleic acid (RNA/DNA), biomimetic, and microbes. Here we look into various types of bioreceptors with their mechanism, examples, applications, advantages, and challenges faced.

### 5.1 Enzymatic biosensors

The first biosensor introduced in 1962 by Clark and Lyons is enzymatic biosensor [77]. Enzyme is the most widely used biorecognition element because of its specificity and catalytic activity. Enzymes are protein that are capable of rushing the rate of reaction (metabolic process) and converting the specific substrate into product without being consumed. This distinctive feature makes enzymes to be used as a bioreceptor in biodevice [78].

There are two types of enzyme biosensors: the substrate and inhibitor enzyme sensor. The former one is to determine the specific substrate of the enzymatic process and the latter one is to detect the substance that blocks the enzyme activity [79]. The selection of exact enzyme immobilized skillfully as a biological element with supportive transducer can give complete detection of biomolecules present in a range of concentrations [80]. It is important to immobilize the enzyme on the electrode. The immobilization can be done using entrapment, adsorption using weak forces, encapsulation, and covalent bonding in order to enhance stability, reuse, and to easily separate product from enzyme.

The most relevant mechanisms used to explain the enzyme actions are lock and key method and induced fit method. In the lock and key method, the enzyme and substrate have rigid shape, i.e., the active site of enzyme can only be locked by a specific type of substrate to yield a product and after the product formation they escape into the surrounding medium. However, in the induced fit model, the enzyme’s active site and substrate are flexible, so that the substrate will have an optimal fit in the active site of enzyme by changing its conformation. Enzyme biosensors have two ways to monitor an analyte: the direct and indirect monitoring. In direct monitoring, the substrate will get attached to the immobilized enzyme along with loosely bonded substrate (co-substrate) to produce the product and these co-substrates or the product produced are measured. For example, in case of glucose, urea, cholesterol, etc. In indirect monitoring, the enzyme activity will be masked by inhibitors by binding to the immobilized enzyme either with free enzyme or enzyme–substrate complex and those inhibitors are being assessed [81].

Hence, the real mechanism in enzyme biosensor is (a) The enzyme bioreceptor will catalyze the substrate into a biosensor recognizable product. For example, H_2_O_2_ in case of glucose biosensor, the GOx catalyzes glucose into H₂O₂ (hydrogen peroxide) and C_6_H_12_O_7_ (gluconic acid). The H_2_O_2_ is easily recognized by specific transducer. (b) Recognition of substrate leads to the change in enzyme activity (enzyme activation or inhibition) in order to measure the substrate or analyte concentration. (c) Analysis of new altered enzyme’s characteristics on the binding of enzyme bioreceptor and analyte.

The simple, most heard, and used enzyme biosensor is glucose biosensor for monitoring blood glucose level with amperometric transducer. An amperometric biosensor detects the current with respect to the time as electrons are exchanged [82]. The enzyme GOx is cheap, easy to obtain, withstand extreme temperature and pH, and have high selectivity for the detection of glucose. The mechanism starts with immobilizing the GOx enzyme which detects and breaks down the beta-d-glucose into H₂O₂ and C_6_H_12_O_7_. The H₂O₂ is electrochemically detected by using an amperometric transducer [83]. The reaction is monitored using tris ruthenium chloride, an oxygen indicator [84].

Urea biosensor uses urease enzyme for the detection of urea in clinical and environmental samples. Urease catalyzes the hydrolysis of urea based on the pH of the solution into NH_4_ (ammonium) and CO_2_ (carbon dioxide) or its ionic forms ( \[{\text{HCO}}_{3}^{\mbox{--}}]\] or \[{\text{CO}}_{3}^{2\mbox{--}}]\] ) [85]. Urea is the final product of the metabolism of nitrogen in human body. And breakdown of urea results in the deposition of toxic product named ammonia (NH_3_). The level of urea in human serum is in the range of 15–40 mg/dL. Based on the ammonium ions produced or change in pH, the transducer will detect the output signal as urease breakdown of the urea. So, with the help of urea biosensor, urea as an indicator, it is possible to know whether the water is polluted with sewage or not. Also in medical application, to know the healthy condition of kidney, urea biosensor is employed [86].

The detection of penicillin or ampicillin (antibiotics) by biosensor uses the beta lactamase enzyme penicillinase, also known as penicillin amidase. The mechanism of bacterial resistance to the beta-lactam ring of antibiotics is credited to the beta-lactamase enzyme. These enzymes hydrolyze and inactivate the beta-lactam ring found in antibiotics, converting penicillin to penicilloic acid. Here covalent bonding and crosslinking are used to immobilize the penicillinase. Furthermore, the use of covalent bonding and crosslinking ensures the stability and durability of the penicillinase enzyme on the biosensor surface. When penicillin attaches to the penicillinase enzyme’s active site, it is broken down into penicilloic acid and other substances. Later, the product is detected by using an amperometric transducer. The penicillin biosensor helps to detect penicillin G in milk of cattle [87], and also identifies the amount of penicillin residues in wastewater to determine the antibiotic discharge on the environment [88]. The major challenges faced by enzyme biosensors are false positive and false negative results due to cross reactivity, recalibration to maintain accuracy, short life span, and extra purification step.

### 5.2 Microbial biosensors

Microbial/microorganism/cell biosensor is based on the biological detection of entire cell/microorganism/cellular component as bioreceptor which is immobilized into a transducer to detect specific analytes. This is a vast category comprising enzymes, non-enzymatic proteins, cellular structures, cells, proteins etc., which can be used as a biorecognition element [89]. Microbes such as bacteria, yeast, and fungi are used as bioreceptors in biosensor by evaluating their metabolism. Here the microbial cell is considered a pool of enzymes, that is when analyte enters the cell, the intracellular enzyme present in it will convert into electrochemically active product. Hence, the metabolic state can be detected by measuring the oxygen level, ionic composition, and carbon dioxide level of the immobilized cell [90].

The mechanism involved in a microbial biosensor is the selection of suitable bioreceptor and biotransducer in order to get the signal and to detect the analytes. The membrane or gel strip will represent the bioreceptor part having the immobilized microorganisms, enzymes, whole cell, Apts, antibodies, phages, etc. These microorganisms are anchored onto the surface of electrochemical transducer, where the target interacts with analyte and converts the changes into a measurable signal. The signal will provide an idea about the concentration of target analyte and by connecting it to a data processor, the output signal can be further characterized [91,92].

For example, Bilitewski and colleagues created a microbial biosensor for measuring short-chain fatty acids from milk. For that, microbe Arthrobacter nicotianae were immobilized in a gel on an electrode surface. Later, the electrochemical measurement of the oxygen consumed by Arthrobacter nicotianae will give its respiratory activity, which can be detected, offering an indirect way of monitoring fatty acid intake [93].

#### 5.2.1 Bacteria-based biosensor

Detection and identification of bacterial pathogens are crucial objectives across diverse sectors such as medicine, food safety, public health, and security. The presence of bacteria in wastewater or in environment can lead to a variety of health conditions such as diarrhea, strep throat, tuberculosis, skin infection, etc. Infectious diseases stand as significant contributors to global morbidity and mortality, resulting in millions of annual deaths and hospitalizations. The methods such as quantitative polymerase chain reaction, colony counting, and cell culture are normally used for the detection of bacteria. Since these methods are time consuming and expensive, bacterial biosensor is proposed for its accuracy and early detection.

Bacterial biosensor uses bacteria as the biosensing element for producing output signal which is proportional to the concentration of analytes. Current research endeavors are primarily directed toward detecting the whole bacteria [94,95]. Notably, impedimetric and optical methods emerge as the predominant approaches in the context of whole bacteria detection. Crafting biosensors for entire microorganisms poses a challenge due to the necessity to detect analytes significantly larger in scale compared to typical molecular analytes such as proteins. Additionally, bacteria exhibit numerous surface epitopes, contributing to potential nonspecific interactions with the biosensor surface. Ligler et al. developed an optical based biosensor for the detection of various bacteria such as *E. coli*, *Salmonella typhimurium*, *Shigella dysenteriae*, and *Campylobacter jejuni* from food and environmental samples [96]. The NRL Array Biosensor was developed to concurrently analyze multiple samples for a range of analytes. The limit of detection (LOD) was established by determining the lowest tested concentration that generated a signal 3 standard deviations above the blank, with values ranging from 2 × 10^3^ to 8 × 10^4^ CFU/mL [96].

#### 5.2.2 Yeast biosensor

Yeast is more similar to humans in terms of molecular and cellular features. Yeast cells, whether in their native state or genetically modified serve as non-specific reporter systems for assessing the toxicity of substances present in various domains such as food samples, environment, cosmetology, and drug. However, toxic compounds exhibit diverse cytotoxic mechanisms; some may not harm yeast cells but can be detrimental to human cells and tissues [97]. Moreover, yeasts employ robust mechanisms for detoxification and expulsion of harmful substances. Specifically, one of the notable systems involved in this process is the pleiotropic drug resistance (PDR) family of ATP-binding cassette (ABC) transporters. The PDR family of ABC transporters in yeasts plays a crucial role in cellular defense against a variety of toxic compounds [98].

*Saccharomyces cerevisiae* is commonly selected as a host due to the extensive array of genetic tools accessible for this particular organism. Throughout history, yeasts have demonstrated excellence as a model for investigating mammalian diseases [99]. Consequently, they prove highly valuable in the development of biosensors directly pertinent to human health. As, an instance, a cellular assay has been established in the yeast *S. cerevisiae* to identify the distinctive protease activity associated with the human cytomegalovirus [100].

#### 5.2.3 Bacteriophage-based biosensor

Bacteriophages or phages are a kind of virus that are used to infect bacteria and identify target bacteria. It is known for long term existence and quick multiplication in host cell, thus making them a valuable tool in diagnostic, therapeutic, water and food quality monitoring. Also, in order to increase sensitivity and selectivity, the bacteriophage can be engineered for a specific bacteria strain [101]. The phage work started in 1915 by Frederick W. Twort by growing Vaccinia virus on agar media instead he found mucoid and glassy colonies of micro cocci a bacterial contamination [102]. In 1917, d’Herelle found out that microbes were able to lyse and kill the bacteria and thus discovered the bacteriophages [103]. Phages are considered as a type of parasite that replicates via bacterial machinery. Main benefits of using bacteriophage as a bioreceptor are that they are pervasive in nature, safe to humans, targets bacteria, can be immobilized onto to transducing device, have long shelf life, and can regenerate.

### 5.3 Advantages and challenges of microbial biosensors

Microbial biosensor is far better than enzyme-based sensor as it does not require any further purification steps, and optimal to pH and temperature. Also, this biosensor is used for monitoring in multiple areas such as food technology, environmental analysis, and clinics. This is a unique type of biosensor in which the material will be extracted from living things. Considering the worldwide abundance of microbes, their simplicity of production, high shelf life, and suitability for immobilization make them ideal for use in biosensing regimens. The innovative simplicity, specificity, and sensitivity of microbe mediated biosensors cannot be overlooked, with applications ranging from the detection of food and waterborne pathogens, military, and homeland defense-related biological threat such as bioweapon can also be detected [104]. Microbial biosensors utilize living microorganisms as the biorecognition element for analyte detection. Table 5 summarizes their key pros and cons.

**Table 5:** Advantages and challenges of microbial biosensor

Advantage	Description	Challenge	Description
High sensitivity and specificity	Microbes can be genetically engineered to respond to specific analytes with high sensitivity due to their complex metabolic pathways	Stability and maintenance	Living organisms require specific growth conditions (nutrients, temperature, pH) which can impact sensor performance and shelf life
Broad range of applications	Microbial biosensors can be designed to detect various analytes, including pollutants, toxins, pathogens, and specific chemicals	Response time	Microbial growth and response to analytes can take time (minutes to hours), limiting real-time applications
Self-regeneration	Some microbes can regenerate, reducing the need for frequent sensor replacement	Limited shelf life	Microbial cultures have a finite lifespan, requiring periodic renewal or regeneration procedures
Cost-effective	Microbial cultivation and sensor development can be relatively inexpensive compared to some complex biosensors	Interferences	Microbial metabolism can be affected by other compounds present in the sample, impacting accuracy
Environmental resilience	Some microbes can tolerate harsh environmental conditions, making them suitable for field applications	Safety concerns	Genetically modified microbes raise biosafety concerns, requiring proper containment measures

There are numerous cons and challenges associated with microbial biosensor in terms of their stability, selectivity, LOD, and so on. Microbial biosensors often face challenges related to poor selectivity due to the lack of specificity in cellular responses to substrates. Advances in biotechnology, coupled with the increasing availability of gene knock in and knock out for various microorganisms, enable the genetic engineering of microbes. Another strategy for improving selectivity involves the creation of microbial sensor arrays. Introducing analytes to these arrays results in a unique response pattern, akin to a fingerprint. Employing artificial neural network analysis in conjunction with microbial sensor arrays facilitates the identification of the target compound [105].

## 6 Immunosensors and aptasensors

Immunosensors and aptasensors are two powerful biorecognition elements used in biosensor design, each with its own strengths and weaknesses. Table 6 shows the comparison of immunosensors and aptasensors.

**Table 6:** Features of immunosensors and aptasensors

Feature	Immunosensors	Aptasensors
Biorecognition element	Antibodies	Apts (single-stranded DNA/RNA oligonucleotides)
Target recognition	Highly specific to a specific antigen epitope (region on a molecule)	Can be tailored to recognize specific target molecules (proteins, small molecules, toxins)
Development	Requires isolation and purification of antibodies from animals (immunization) – More time-consuming and expensive	Apts are selected *in vitro* (in a test tube) using a process called Systematic evolution of ligands by exponential enrichment (SELEX) – faster and more cost-effective development
Stability	Antibodies are proteins and can be susceptible to denaturation by changes in pH, temperature, or harsh chemicals	Apts are more stable than antibodies – Can withstand wider ranges of temperature, pH, and some organic solvents
Regeneration	Antibodies are not easily regenerated – requires new antibody production for each sensor	Apts can be regenerated by heating and cooling cycles or denaturing and renaturing procedures
Specificity	Highly specific but can have cross-reactivity with similar molecules	Can be highly specific but development may require more optimization for complex targets
Size	Antibodies are relatively large molecules – may limit miniaturization of biosensors	Apts are smaller than antibodies – can facilitate development of miniaturized biosensors
Applications	Widely used in medical diagnostics (pregnancy tests, immunoassays) – environmental monitoring (toxin detection) – food safety testing (pathogen detection)	Gaining traction in medical diagnostics (cancer markers) – environmental monitoring (pollutant detection) – biosecurity applications (biothreat detection)

### 6.1 Immunosensors

Immunosensors are biosensors based on antibodies in order to find specific antigens i.e., an affinity-based biosensing device. Or it can also be stated as a type of biosensor having bioreceptor as antibodies or antigens. The journey of immunosensor began in the 1950s, since then onwards it is used in the clinical diagnostic purposes [106,107]. Antibodies/immunoglobulins (*I*_g_), characterized by a “Y” shape and composed of two heavy (H) and two light (L) chains. In certain cases, human antibodies have a third chain called the joining (J) chain giving it a dimeric or pentameric configuration. Each chain, whether heavy or light, consists of constant and variable regions. The variable part is unique to the associated antigen, allowing antibodies to exhibit a highly selective and specific binding capability. Utilizing the antigen as a bioreceptor, an immunosensor benefits from the antibody’s specificity, stability, and adaptability to effectively interact with the corresponding antigen [107].

Immunosensors commonly employ the solid-phase immunoassay principle, where stable substrates immobilize both antigens and antibodies. This arrangement facilitates the interaction between antigen and antibody specifically at the interface between the solid and liquid phases [108]. Like enzymes, antibodies are immobilized in the transducer using adsorption method. Antigen and antibody interaction on the electrochemical transducer will give rise to electrical signals which can be monitored by connecting to a signal processor [109]. The utilization of electrochemical immunosensors is quite effective for sensitive and targeted identification of the intended analyte. They need minimal sample volumes, inexpensive, straightforward, and simple. In recent years, there has been an increase in interest in electrochemical immunosensors, which effectively integrate immunoreactions with electrochemical transducers [110]. The principle of electrochemical immunosensors involves directly immobilizing antibodies and then adding the targeted analytes for electrochemical measurement. Different immobilization procedures are suitable depending on the analyte’s molecular size. This method works well for analytes with high molecular sizes. For smaller molecules, because of their unstable immobilization onto the electrode surface, direct immobilization of antibodies may result in structural alterations for smaller analyte molecules. As a result, inaccurate results may be found. To overcome this, it is used along with nanomaterials to prevent the functional group from possibly fading during the direct immobilization of analytes [111].

Yuan and colleagues have presented research on potentiometric immunosensors that have been able to detect the hepatitis B surface antigen. This approach involves immobilizing the biorecognition element on the platinum (Pt) electrode system, which is hepatitis B antibodies coupled with AuNPs and polyvinyl butyral [112]. They showed that in response to the hepatitis B surface antigen (HBs Ag), the immobilized hepatitis B surface antibody demonstrated direct electrochemical behavior. Immunosensor due to its selective property, simple, cost-effective, and rapid determination of analytes is used in various fields such as clinical diagnosing, food control, and environmental monitoring. Immunosensor’s primary drawback is their inability to determine small-molecular weight analytes [75,113]. Additionally, the use of labelled antibodies results in an insulating layer on the modified electrode that prevents electron transport and increases impedance because of the unique interaction between the antibody and antigen. This can be modified by using electrochemical impedimetric immunosensors, which get rid of the need for enzyme labelling and catalysis [114].

### 6.2 Aptasensors

The biosensors using the Apts as a bioreceptor are commonly known as aptasensors. Apts are highly specific and affinity-binding single-stranded DNA (ssDNA) or RNA molecules. The usage of Apts enables efficient immobilization at high density and is appropriate for the quick and simple detection of proteins due to their small size (30–100 nucleotides) in comparison to antibodies and enzymes [115]. Aptasensors can also be classified under biomimetic sensor as it mimics the role of a natural biosensor. That is, aptasensors are artificial nucleic acid strands able to recognize peptides, amino acids, proteins, etc. The term “aptamer” was first documented in the early 1990s. Apts exhibited unexpected adaptability when compared to other bioreceptor components, yet, they behaved more like antibodies [116,117]. They are frequently produced via SELEX, an Apt selection process that involves selecting genes primarily at random from different oligonucleotides. SELEX employs two steps, i.e., by using a polymerase chain reaction, the natural oligonucleotides are first amplified to the required concentration. Then, this amplified pool interacts with particular targets, and the participating oligonucleotides are utilized in the initial phase of the subsequent SELEX round [118,119].

Apts are mainly classified into two types: peptide Apts and nucleic acid Apts. Peptides are polymers composed of amino acids that are either naturally occurring or artificially synthesized, and they are built in a manner akin to that of proteins. Peptide libraries are collections of diverse peptides with various amino acid sequences that can be screened to identify those with high affinity for a specific target. By systematically testing different peptides, researchers can discover new recognition receptors or optimize existing ones for enhanced selectivity and specificity. This approach allows for the development of peptide-based therapeutics, diagnostics, and biomaterials with tailored properties and functionalities. When it comes to conformational and chemical stability, peptides have several advantages over proteins, including a significantly lower likelihood of denaturation [120].

Nucleic acid Apts are further classified into RNA and DNA Apts. DNA Apts are most prominently known for their durability and ease of synthesis with high purity and repeatability, facilitating the development of biosensors. Conversely, biosensors utilizing RNA Apts often face limitations in repeated detections due to their susceptibility to degradation by endoribonucleases present in biological environments [115]. These biosensors function by immobilizing DNA probes on an electrode surface, enabling specific target DNA sequences to bind and produce a signal. The integration of electrochemical techniques with DNA analysis has significantly transformed genetic diagnostics and holds promise for improving healthcare systems globally. For example, the anti-thrombin Apt, which has a specific sequence of 15 nucleotides and is labeled with fluorescein isothiocyanate, was immobilized on a microscope cover slip using covalent bonding. The fluorescence anisotropy of the immobilized Apt was then observed using evanescent-wave-induced fluorescence. This allowed for the identification of protein binding, specifically to thrombin. To remove the bound thrombin and rejuvenate the sensor, the Apt-coated glass slide was rinsed with a phosphate buffer solution followed by guanidinium hydrochloride treatment. Finally, the sensor was brought back to its original state through phosphate buffer solution equilibration [121]. Compared to many other recognition components, such as antibodies and enzymes, Apts provide a number of advantages. They have a great degree of selectivity and specificity for almost any material, from microscopic molecules to whole cells, proteins, and peptides, and may be generated *in vitro* without the requirement for animal hosts. When it comes to handling the conditions that arise during sample collection, aptasensors will be more resilient and flexible. It frequently performs better in terms of stability than other biological substances and is commercially produced in its pure form [90].

## 7 Nanomaterial-based biosensors

Nanoparticles (NPs) smaller than 100 nm in at least one dimension are considered nanomaterials. Nanoscale materials are used in nanomaterial-based biosensors, which have special qualities that improve biosensing devices’ sensitivity and functionality. These nanoscale materials possess unique properties such as high surface-to-volume ratio and enhanced electrical conductivity. These qualities enable the biosensors to detect and analyze biomolecules with higher precision and accuracy, making them valuable tools in various fields including medical diagnostics and environmental monitoring. Also, it has been discovered that the use of nanomaterials may improve the performance of biosensors, resulting in higher sensitivities and lower limits of detection by several orders of magnitude [122]. Nanomaterials have revolutionized biosensor design due to their unique properties at the nanoscale. Table 7 summarizes their key features and applications.

**Table 7:** Key features of nanomaterial-based biosensors

Feature	Description
Nanomaterials used	Carbon nanotubes (CNTs) [123], graphene [124], metal NPs (AuNPs, PtNPs) [125], metal oxides (ZnO, TiO_2_) [126], quantum dots (QDs) [127].
Advantages	High surface area: Enables greater biomolecule immobilization, enhancing sensitivity
	Tailorable properties: Size, shape, and surface chemistry can be tuned for specific target interactions. Improved conductivity: Can enhance electron transfer efficiency in electrochemical biosensors
	Optical properties (QDs): Enable fluorescence-based detection with high sensitivity
Applications	Medical diagnostics: Early disease detection (cancer markers), monitoring biomolecules (glucose, hormones)
	Environmental monitoring: Detection of pollutants (heavy metals, toxins) in air and water
	Food safety testing: Identifying pathogens and contaminants in food products. Biosecurity applications: Rapid detection of biothreat agents
Challenges	Biocompatibility: Some nanomaterials may raise concerns regarding their interaction with biological systems. Aggregation: NPs can clump together, reducing their effective surface area and hindering performance. Cost: Production of some nanomaterials can be expensive, impacting sensor affordability

From 1981 onward, nanotechnology has witnessed a rapid advancement. The most researched ones are graphene (G), CNTs, AuNPs, and photonic crystals because of their distinctive characteristics among all other nanomaterials. They were great options for biosensor applications because of their significant signal amplification effect, high surface energy, outstanding biocompatibility, and high stability [128,129]. For example, in order to increase the precision and sensitivity of glucose sensors, nanotechnology has been applied. Glucose sensors can be developed via the use of nanofabrication methods or macro- or microscale components. These enhanced systems have increased catalytic activity and greater surface areas, which result in bigger currents and faster reactions. Furthermore, due to their small size, these sensors may be able to evade the immune system’s foreign body reaction and thus have longer lives [130].

Three elements make up nanomaterial-based sensors: one or more nanomaterials, a specificity-enhancing bioreceptor, and a signal transduction mechanism that relays the analyte’s presence [131]. Mainly carbon-based NPs (graphite, CNTs) and metal-based NPs (gold, silver) are incorporated in biosensors. CNTs and graphenes, due to their high electrical conductivity, high thermal conductivity, and strong mechanical strength, are considered a great option for detecting environmental pollutants such as heavy metal ions, pesticides, toxic gases, and so on. Reduced graphene oxide (GO) is the primary graphene form used in electrochemical sensing [132]. It is readily obtained from graphite using the Hummers method. For instance, Liu and Lu developed a colorimetric Pb^2+^ biosensor by introducing Pb^2+^-specific DNAzymes as the target bioreceptor and DNA-functionalized AuNPs as the signaling element [133]. When Pb^2+^ is present, the cooling process causes the enzyme to cleave the substrate strand, preventing the formation of NP aggregates and producing a red hue. Therefore, Pb^2+^ may be identified and measured by looking at the color of the sensor. Miniaturization, mobility, and quick signal response times are just a few of the breakthroughs in sensor design made possible by nanomaterials. Nanomaterials are very sensitive to changes in surface chemistry due to their high surface area-to-volume ratios and ease of surface functionalization. This allows nanosensors to attain incredibly low detection limits. Furthermore, the small size of nanomaterials allows for their integration into various devices and systems, enhancing their versatility and applicability in different fields such as healthcare, environmental monitoring, and food safety. Additionally, the quick signal response times of nanosensors enable real-time monitoring and detection of target analytes, providing valuable insights for timely decision-making and intervention [38].

## 8 Design and fabrication of bioelectrochemical biosensors

Bioelectrochemical biosensors involve a meticulous process integrating biological recognition elements with electrochemical transducers. Table 8 outlines the key steps in their design and fabrication.

**Table 8:** Summary of key stage for design and fabrication of biosensors

Stage	Description	Consideration
Target analyte and detection mechanism	Identify the target molecule and choose a suitable detection strategy (enzyme reaction, antibody binding, etc.) based on the analyte’s properties	Specificity of biorecognition element
		Feasibility of electrochemical readout for chosen mechanism
Electrode design and material selection	Design the electrode geometry and choose a conductive material (gold, platinum, carbon-based) considering surface area, biocompatibility, and target interaction	High surface area for biomolecule immobilization. Compatibility with chosen detection method
Biorecognition element immobilization	Attach the chosen biomolecule (enzyme, antibody, etc.) to the electrode surface using appropriate methods (physical adsorption, covalent bonding)	Orientation and stability of biomolecule
		Maintaining bioreceptor activity
Transducer selection and integration	Choose the appropriate electrochemical technique (amperometry, potentiometry, etc.) based on the detection mechanism and integrate it with the electrode system	Sensitivity and selectivity of chosen technique. Compatibility with overall sensor design
Calibration and optimization	Test the sensor performance with known analyte concentrations to establish a calibration curve and optimize parameters (buffer conditions, applied potential) for optimal sensitivity	Linearity of response. Minimizing background noise
Miniaturization and integration	In some cases, miniaturize the sensor design for portability and integrate necessary components (electronics, microfluidics) for a complete sensing platform	User-friendliness and portability
		Maintaining sensor performance in a miniaturized format

### 8.1 Electrode materials and modifications

Fossil fuel is the main source of energy. The energy derived from fossil fuel is finite and non-renewable, making it unable to keep up with the world’s growing energy needs [134]. By the year of 2024, oil and coal production are predicted to decrease significantly, further exacerbating the energy crisis. As a result, it is crucial for countries to invest in alternative and renewable sources of energy to ensure a sustainable future [135]. Hence, the sustainable solution for this crisis is the development of BES commonly the MFC system primarily using anode electrode and cathode electrode to make the energy.

Electrodes are a mediator, i.e., a non-metallic material capable of conducting electricity from two or more chambers containing different sample solutions. In BES, electrodes detect the chemical energy and converts into electrical energy. Electrode materials are materials mainly used to construct or manufacture the electrodes. This can be metals, carbon, metal oxides, composite materials, carbon allotropes, and so on and may vary with function and purpose of the MFC. The most favored electrodes for anodes and cathodes are graphite rods, carbon cloths, carbon papers, stainless steel mesh, graphite mesh, tungsten carbide, platinum, nickel oxide, titanium, and graphite foils. The more usage of carbon-derived materials in past research of MFC is due to its affordable cost, superior conductivity, biocompatibility, and wide surface area. But after using these electrodes continuously, it has drawbacks of membrane fouling and low capacity for electron transfer [135]. This led to the modification of electrodes by natural [136], synthetic, and chemical [137] ways. Nanostructural tool and polymers have been integrated with the normal electrodes in order to make modifications in electrodes and to expand the power generation in MFC, majorly by using graphene, polyaniline (PANI) CNTs, poly-*N*-isopropylacrylamide (PNIpam), polycrystalline carbon rod, carbon fiber veil, and graphite fiber brush. In a study conducted by Xie et al. in 2012, they used a modified electrode made of a CNT–sponge composite as anode and a platinum-coated CNT sponge as cathode. They observed a maximum power of 1.24 W m^−2^ during the treatment of wastewater [138]. Using some natural anode modifications such as layered corrugated carbon, granular electrodes, King mushroom carbon showed good results in power generations. A study shows that stirring granular activated carbon (GAC) in an anode compartment increases the power density by 17%. Activated carbon granules help in capturing the electrons produced by the biofilm and store them. The biofilm on GAC acts as a capacitor, discharged when in contact with the current collector, resulting in 951 and 813 mW/m^2^ power density while stirring and without stirring, respectively [139]. Table 9 shows various modified electrons and their generated power while treating wastewater.

**Table 9:** Various modified electrodes and performance of BES

Anode	Membrane type	Cathode	Source	Max power/current	Reference
Stainless steel fiber felt	Ultex	Carbon felt	Effluent from MFC	2,142 mW/m^2^	[140]
Bamboo-NCNT	Cathode exchange membrane (CEM)	Carbon brush	Aerobic and anaerobic sludge	1,040 mW/m^2^	[141]
SDS-EG-MWCNT	NA	MWCNT-PTFE	Anaerobic sludge	1,640 mW/m^2^	[142]
GPG composite	Nafion 117	Graphite electrode	Geobacter sulfurreducens	3,804 mA/m^2^	[143]
Carbon felt		Carbon fiber	Sediment and seawater	8.5 mW/m^2^	[144]
Sugar-urea carbon foam		Carbon fiber	Sediment and seawater	190 mW/m^2^	[144]
Magnetite entrained sugar urea carbon foam		Carbon fiber	Sediment and seawater	67 mW/m^2^	[144]

### 8.2 Conductive materials

To enable electron transport from the microbes to the electrode, MFCs usually use conductive materials in their fabrication. These substances play a critical role in maintaining effective electron transfer processes and improving the electrode’s conductivity. The mainly used conductive materials in MFC are carbon-based, metal-based, conductive polymer (CP)-based, and composite-based material.

### 8.3 Carbon-based material

Materials based on carbon are frequently chosen because of their exceptional physical qualities and relative light weight [145]. Carbon-based materials are made up of carbon atoms arranged in various structures. For example, diamond is a carbon-based material that consists of carbon atoms arranged in a crystal lattice structure. Fullerenes and c60 are arranged in cages made up of hexagonal and pentagonal rings, CNTs are made up of carbon atoms arranged in a cylindrical structure/tubes, and graphene is composed of a single layer of carbon atoms arranged in a hexagonal lattice/sheets [146]. The most favored carbon-based materials are graphene (G), CNTs, carbon nanofiber, fullerenes, and GO due to their unique property such as active surface area, mechanical strength, and conductivity. Graphene is an allotropic, two-dimensional form of carbon made up of a single sheet of carbon atoms organized in a hexagonal lattice. Andre Geim and Konstantin Novoselov made the discovery of graphene in 2004, which is composed of graphite sheets that are only one atom thick [147]. The characteristic known as electronegativity of carbon atoms allows them to naturally form covalent bonds with a wide variety of other elements, making them excellent at forming covalent bonds with other atoms. Graphene-based materials can also be used as nanoprobes for biosensors. For example, GO contains groups having oxygen on its surface. Hence, during reduction of this group, it produces distinct signals. So, GO can be utilized as a label in an electrochemical biosensor. Additionally, Akhavan et al. used reduced graphene nanowalls (RGNWs) for the creation of an electrochemical biosensor with extremely high resolution. The oxidation signals of the DNA bases (AGTC) were found using differential pulse voltage. It implies that the RGNW electrode outperformed glassy carbon, graphite, and reduced graphene nanosheet electrodes in terms of performance. After 100 DPV scans, the RGNW electrode showed outstanding stability with just a 15% variance in oxidation signals. With the RGNW electrode, the lower detection limit for dsDNA was around 9.4 zeptomolar (zM), or about 5 dsDNA molecules per milliliter [148]. The creation of carbon-based electrochemical sensors has drawn the attention of several researchers due to graphene’s high electron transfer rates in diverse mediums [149,150].

Iijima, a Japanese scientist, discovered CNTs in 1991 as one of the allotropic modifications of carbon. Rolls of graphene sheets stacked in cylindrical or tubular shapes with a diameter of several nanometers are the characteristic feature of CNTs. The use of CNTs as electrode surface modifiers is growing, partly because CNT-modified electrodes perform better than other electrodes such as Au and Pt and have greater conductivity than graphite [149]. Because of their large surface area and nanoscale diameter, CNTs can effectively transfer electrons between protein redox sites through direct electrochemical communication. The modification in CNT has showed large surface area, low voltage, and quick electrode kinetics, low LOD, and a quick response [151]. Britto et al. created the first CNT-based sensor with increased reversibility to examine dopamine oxidation [152].

### 8.4 Metal-based material

Many metals, including copper, gold, and titanium, have been explored to be used as MFC anode electrodes. Metal-based materials are comparatively better electrical conductors than carbon-based materials, but their corrosion resistance is less favorable. So, the majority of these metals were not appropriate. But stainless steel showed remarkable result [153]. Also, metals are used along with nanomaterial for good results.

### 8.5 CPs

Conducting polymers are materials made of organic polymers that are electrically conductive. A variety of techniques, such as chemical oxidation, electrochemical polymerization, vapor phase synthesis, and hydrothermal, were used to create conducting polymers. In their pure form, conducting polymers often exhibit poor optical and electrical conductivity; however, doping them with appropriate substances, such as donors or acceptors of electrons, can give them exceptional qualities. The most commonly used CPs are PANI, polypyrrole (Ppy), polythiophene (PT), and PNIPam [154]. The low cost, scalability, and ease of manufacturing of conducting polymers, along with their high chemical specificity, tunable transport properties, and surface area, make them great choices for electrochemical sensing applications. Ppy is ecologically safe and electrically conductive, it is mainly used in composite form in MFC. Wang and his coworkers used anode that was treated with PANI and found improvement in the bacterial cell’s adhesion and enhanced its electrochemical activity [155]. PANI is a biocompatible, readily produced, and reasonably priced material; yet, when doped on electrodes, its shrink-swell capability compromises its stability, lifetime, and ability to transmit electrons [135].

### 8.6 Surface functionalization and immobilization methods

**Figure 4:**
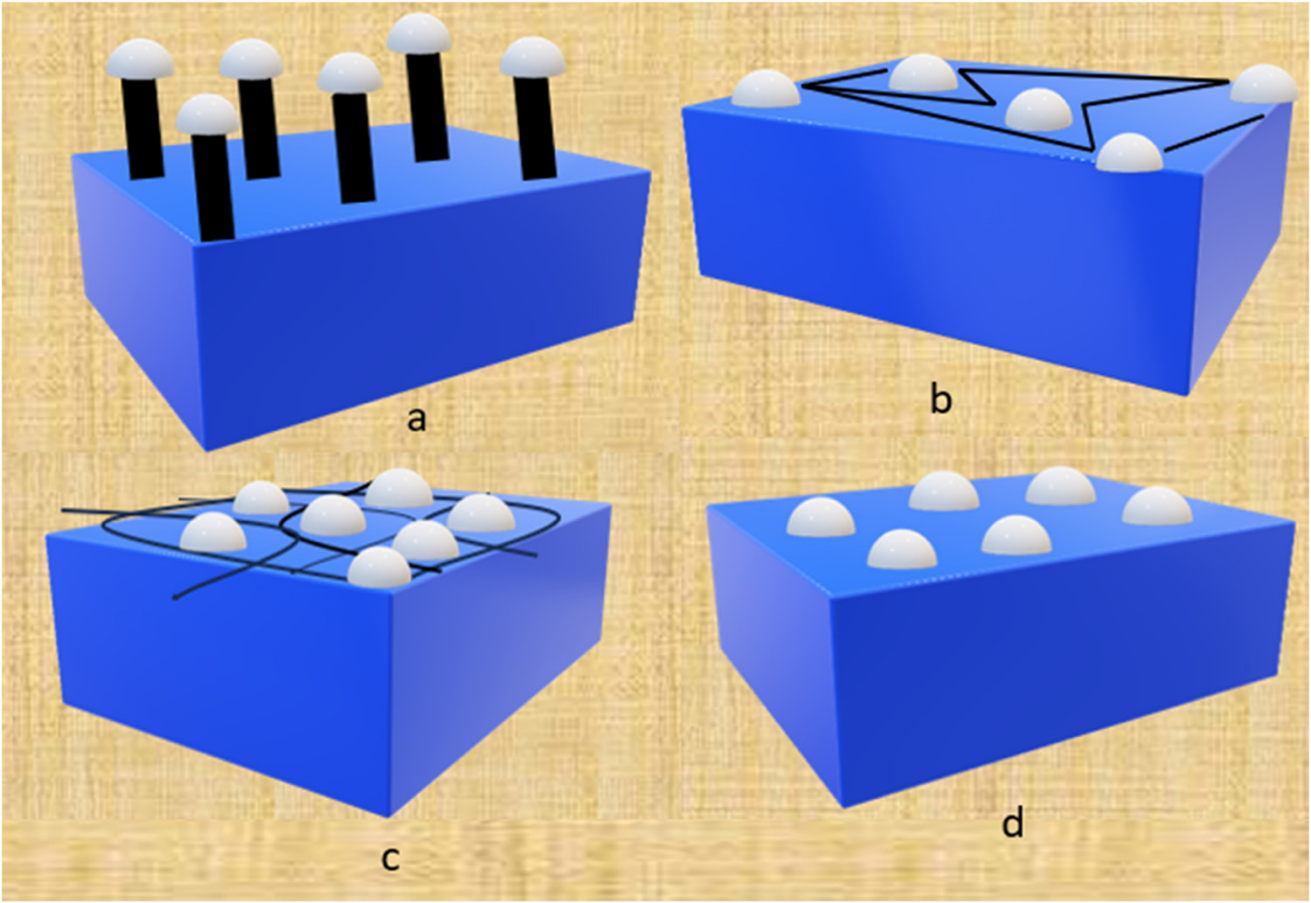
Different types of immobilization methods used in a biosensor: (a) Covalent bonding, (b) cross linking, (c) entrapment, and (d) physical adsorption.

Surface functionalization is a process of changing or modifying the electrode material’s surface characteristics in order to enhance its function in wide application whereas immobilization methods are used to fix the bioreceptor with suitable transducer. The biomolecules that are immobilized on a transducer part of a biosensor should have to maintain a constant structure, function, and biological activity for longer and stable life. Hence, immobilization is a key part for a biosensor for sustaining its long-term life and to do operations accurately and in a biocompatible way [156]. The immobilization techniques that are mostly used in the making process of a biosensors are categorized into physical methods (entrapment and adsorption) and chemical methods (cross linking and covalent bonding). Adsorption is the attachment or fixing of a biomolecule into the surface of a transducer by using non-covalent forces (weak bonds) such as van der Waals bonds, hydrophobic bonds, hydrogen bonds, and electrostatic bonds. It is a simple type of immobilization technique. Adsorption can be done by various methods such as physical adsorption [157], layer-by-layer [158], electrostatic interactions [159], electrochemical doping [160], and lipidic microenvironment [161]. In the research conducted by Tang et al., the amperometric biosensor was developed in which he and his colleague’s used adsorption as immobilization method to adsorb GOx in platinum-modified CNT electrode. Furthermore, the electrode resultant is a good electrocatalytic activity with a determination range of 0.1–13.5 mM, a short response time of under 5 s, a large current density of 1,1760 mA m^−2^, high sensitivity of 91 mA M^−1^ cm^−2^, and stability of 73.5% even after 22 days [162]. Entrapment is a method of immobilizing enzymes in a 3D matrices or scaffold in its native form without harming its biological activity. The 3D scaffold can be a gel or polymer (Figure 4).

The various techniques utilized in entrapment methods are electropolymerization, photopolymerization, silica sol–gel, polysaccharide-based gel, and carbon paste electrode. For instance, to create an amperometric biosensor for the detection of cholesterol, an electropolymerization-based entrapment methodology was utilized to entrap the enzyme cholesterol oxidase in poly(3,4-ethylenedioxypyrrole). It maintained its reactive activity for 20 days without any loss of catalytic activity, along with a response time of 150 s and a detection limit of 4 × 10^−4^ M [163]. Cross-linking is a type of irreversible linkage, forming a complex network by linking with each other without any support. It forms intramolecular cross linkages with the enzyme molecules with the help of reagents [164]. The reagents used for cross linking can be glutaraldehyde or other glyoxal or hexamethylenediamine or bovine serum albumin (BSA), in which glutaraldehyde is the economically feasible one [156]. For sensing blood glucose level in fish, a needle biosensor was developed. In addition, glutaraldehyde was used to induce a cross linking between enzyme GOx and BSA. For the purpose of continuous monitoring, they implanted the sensor into the fish’s eye and the glucose concentrations in the eyeball’s scleral interstitial fluid were regularly monitored between 3.9 × 10^−3^ and 2.3 × 10^−2^ M [165].

Covalent bonding is a chemical irreversible type immobilization method using bond formation between support (solid) and the sensing molecule. This is the most popularly used immobilization methods because of its stability of the bonds which prevents the escape of enzymes into the surrounding environment, making it a long term stable biosensor [156]. In order to determine the cholesterol level of a solution, a cholesterol biosensor has been developed with the cholesterol oxidase (ChOx) covalently immobilized with self-assembled monolayer (SAM) of *N*-(2-aminoethyl)-3-aminopropyl-trimethoxysilane using carbodiimide [166]. The amount of cholesterol utilized in a solution was detected by using absorption method (UV-Visible technique) with an enzyme activity of 1.81 × 10^−3^ U cm^−2^ and a shelf life of 10 weeks.

## 9 Miniaturization and microfabrication of BES biosensors

Miniaturization and microfabrication are transforming BES design by creating compact, portable, and often disposable biosensors. Table 10 summarizes the key benefits, techniques, and considerations.

**Table 10:** Key features of miniaturization and microfabrication of BES biosensors

Benefit	Description	Technique	Description	Consideration
Portability and wearability	Miniaturized BES can be integrated into wearable devices for continuous health monitoring or implantable for real-time biomolecule detection	Thin-film deposition	Techniques such as sputtering or evaporation deposit thin films of electrode materials (gold, platinum) on microfluidic platforms	Compatibility of material with biological components. Surface properties for biomolecule immobilization
Microfluidics integration	Microfluidic channels enable precise control over sample volume, reducing reagent consumption, and facilitating automation	Photolithography	This technique uses light-sensitive polymers to pattern electrodes and microfluidic channels on a substrate	Channel design for efficient flow and minimal dead volume
Mass production	Microfabrication techniques allow for high-throughput production of BES devices, reducing costs	Micromachining	Processes such as micromilling or laser ablation can create microfluidic channels and chambers within a substrate	Precision and resolution to achieve desired miniaturized features
Integration with electronics	Miniaturized electronics can be integrated for signal processing, amplification, and data transmission	Cleanroom techniques	Fabrication often occurs in cleanroom environments to minimize contamination	Cost and expertise required for cleanroom facilities

Miniaturization can improve response times due to shorter diffusion distances of analytes to the biorecognition element. Microfabricated BES devices can be disposable, reducing waste, and cost per test. Biocompatibility considerations are crucial as miniaturized BES may come in closer contact with biological tissues.

### 9.1 Lab-on-a-chip and wearable biosensors

**Figure 5:**
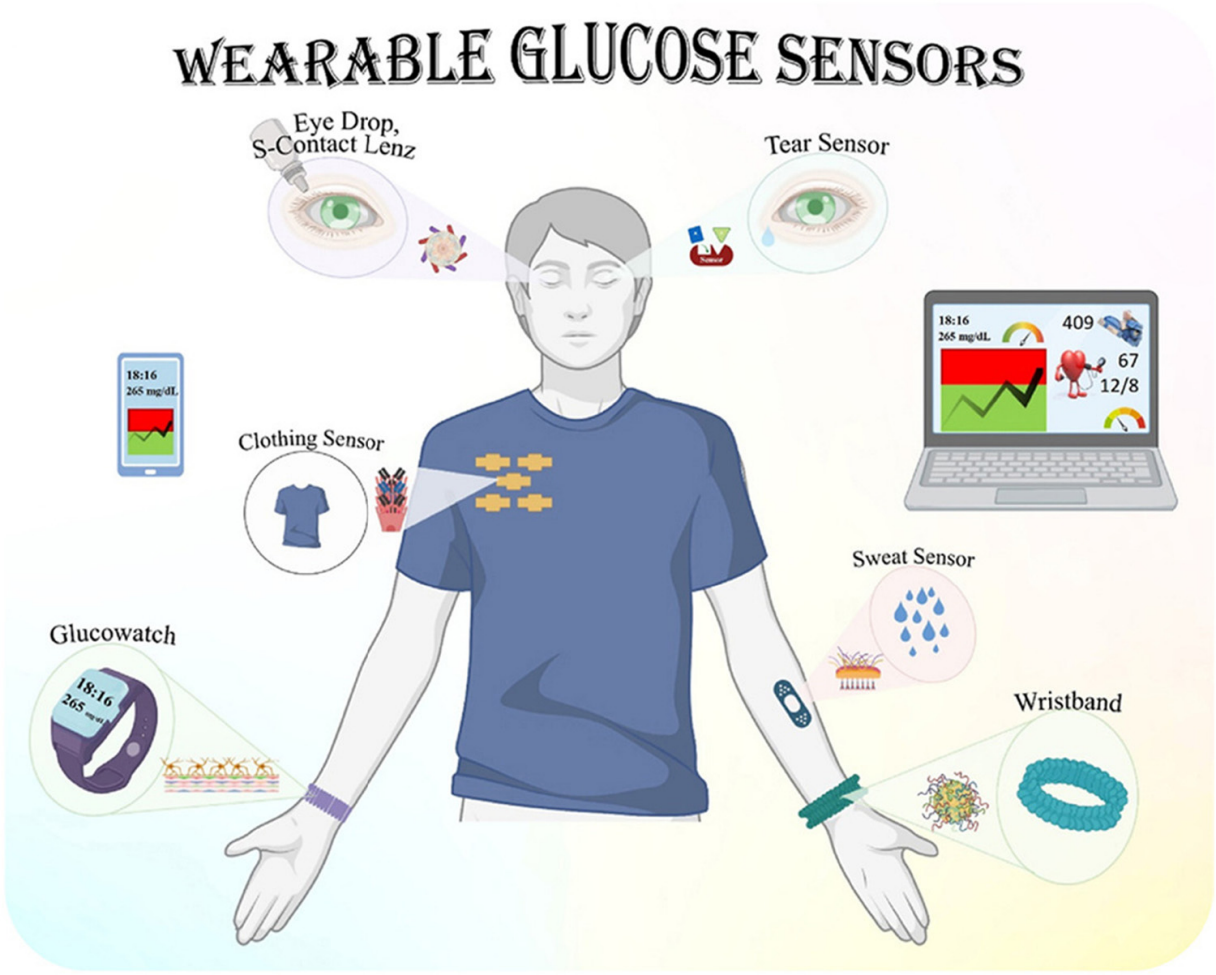
Different types of wearable glucose biosensors used by a patient. To overcome the uncovered needs such as inexpensive, real-time identification of diabetes, early digital health awareness, and limits for intravenous medication [155].

Wearable sensors have witnessed a significant surge in both research and commercialization in recent times. However, advancements in wearable sensor technology have been characterized by a combination of successes and challenges. The predominant commercial progress has been achieved through the intelligent adaptation of established methods in mechanics, electronics, and optics for body measurements. The growing number of diabetes patients worldwide and the growing need for portable diagnostic tools are projected to drive considerable growth in the biosensor market. For example, wearable sensors are crucial in glucose monitoring as shown in Figure 5 to overcome the uncovered needs such as inexpensive, real-time identification of diabetes, early digital health awareness, and limits for intravenous medication. The creation of trustworthy and intuitive wearable sensors for continuous tracking of glucose is emphasized. The goal of these next-generation sensors is to offer continuous surveillance, which is essential for managing diabetes successfully [167]. The primary difficulties encountered are as follows: (i) Establishing a standard for the distribution of insulin: standardizing medication or insulin dosages to guarantee that patients receive the appropriate dosage based on their blood glucose levels is a major difficulty. (ii) Instantaneous detection: Effective diabetes management requires the use of real-time, low-cost detection technologies to track elevated blood sugar levels. (iii) High awareness of digital well-being: Controlling the negative effects of injectable drugs and avoiding problems requires early digital health knowledge.

As a response, researchers have created wearable patch devices, which employ sensitive bioelectronics to precisely measure ultra-trace levels of glucose in perspiration in real time. To efficiently collect perspiration straight from the skin’s surface, a wearable sensor patch that expands with the body’s movements is being created. Sweat glucose is measured with ease due to the small, detachable strip design. There is growing interest in multifunctional devices that can inject insulin via tiny needles and monitor blood sugar levels. Through microneedles affixed to the adhesive patch, these devices assess glucose levels accurately through perspiration and subsequently provide an insulin dosage that is perfectly adjusted [168]. Presently, diagnostic tools are available for almost every analyte that a physician might wish to assess in a patient. Regrettably, these tools are not wearable and still predominantly rely on a blood draw and traditional bench-top assay techniques [169].

The Lab on a Chip represents a continuous sensor designed for the next-generation frontiers, building upon numerous breakthroughs previously documented. According to a recent study, nanoscale phospholipid polymers are revolutionary for many kinds of applications, such as micro- and nanofabricated fluidic systems. A derivative of nanoscale phospholipid polymers, 2-methacryloyloxyethyl phosphorylcholine (MPC) polymers have special qualities that make them excellent choices for a wide range of biological applications. They can function as extracellular matrices, scaffolds for cell culture, and imitate synthetic cell membranes. Poly(MPC-*co*-BMA), poly(MPC-*co*-*n*-butyl methacrylate-*co*-*p*-nitrophenyloxycarbonyl-poly(ethylene glycol)methacrylate [PMBN], and copolymers generated from MPC are examples of recently discovered MPC polymers. These characteristics include the capacity to pass across plasma membranes without harming cells, and the absence of fouling. These polymers can also be used as functional biomaterials in a variety of biological domains, including drug delivery systems and lab-on-a-chip biosensors [170].

As another illustration, polydimethylsiloxane (PDMS)-based enzyme-linked immunosorbent test (ELISA) is a lab-on-a-chip device that was created with PMBN polymer coatings. The PMBN polymer coating is accomplished via electrospray deposition on a bottom glass surface with a designed gold electrode, and microchannels and reaction microchambers on an elevated PDMS-based plate. Research has demonstrated that microchips coated with PMBN polymers structured like nanospheres display reduced nonspecific signals of human thyroid stimulating hormone as compared to conventionally coated microchips [171].

In the 1980s, the broader public started to feel the influence of wearable sensors. But still, it is on the developing phase. The meaningful exploration of wearable chemical sensors took a considerable amount of time before being commercially introduced. An illustrative instance dates back to 1962 when Leland Clark and Ann Lyons from Cincinnati Children’s Hospital developed the initial glucose enzyme sensing electrode. However, the pursuit of a non-invasive wearable sensor took even longer. A significant historical case is exemplified by the introduction of the GlucoWatch product by Cygnus in 2002. The GlucoWatch represented a notable achievement in non-invasive biosensing of glucose for individuals with diabetes. The device employed two gel pads on the skin, cyclically applying DC potential to extract both interstitial fluid and glucose through reverse iontophoresis [172].

## 10 Applications of bioelectrochemical biosensors

### 10.1 Medical and healthcare applications

These days, biosensors have gained too much attention in many domains, such as clinical analysis and illness diagnosis [173]. The capacity to identify biomolecules linked to illness is essential for both the creation of new drugs and the precise diagnosis of existing illnesses. Because of its high susceptibility, low cost, and compact size, the electrochemical biosensor is the most often utilized form of biosensor in medical and health care services, particularly in combinatorial tests [174]. Integrating a biosensor into an electrochemical biosensor is essential for maximizing its potential and sustaining its technical integrity, sample solution availability, and appropriate sample management [175].

### 10.2 Glucose monitoring for diabetes management

The Diabetes Atlas [176] published by the International Diabetes Federation (IDF) states that 10.5% of adults aged 20–79 have diabetes, with nearly half of them being unaware that they have the disease. According to IDF forecasts, 783 million individuals, or 1 in 8 of the population, would have diabetes by 2045 – a 46% rise. Diabetes was believed to be the cause of more than four million fatalities. Also, more than 463 million individuals were at risk of acquiring type 2 diabetes, and more than 23.1 million children had type 1 diabetes. Diabetes-related medical expenses have risen to more than $700 billion globally, accounting for 12% of all adult healthcare spending [176].

These led to the generation of inexpensive one-time usable electrochemical biosensor for glucose monitoring. An amperometric sensor having the enzyme GOx, immobilized to the electrodes. A key component of effective diabetic care is routine blood sugar monitoring. The majority of blood sugar monitoring techniques require a blood sample. Numerous gadgets may be used at home for blood sugar monitoring, e.g., using an instrument to puncture the skin, and then obtain a blood sample. A basic instrument known as a glucometer, or glucose meter, calculates the amount of sugar present in a sample of blood [177]. There are three generations of glucose biosensor available in order to make its design simple and advanced working efficiency. Enzymes (GOx) were utilized as biorecognition components in the first generation of biosensors developed in the 1970s [178]. Enzymes were mounted on a transducer surface in order to measure changes in physicochemical parameters such as pH or electrical current by catalyzing an interaction with the target analyte. Enzyme stability, longevity, and interference from other substances were the difficulties faced by the biosensor. These challenges were addressed in the second generation of biosensors in 1990s by incorporating immobilization techniques and replaced oxygen with redox mediator (ferrocene derivative) to enhance enzyme stability and prolong their lifespan [179,180]. Additionally, advancements in CPs, NPs, and nanotubes have helped to improve stability and lowered reaction times, which made them valuable for environmental monitoring, food safety, and medical diagnostics. Also, minimized interference from other compounds made the biosensors more reliable and accurate in detecting target analytes [178]. Since the 2000s, third-generation biosensors have been developed using cutting-edge technologies such as nanomaterials, nanoelectronics, and biorecognition components. By electronically connecting the enzyme to the electrode, ruled out the use of a mediator, allowing for a straightforward electron transfer [181]. The integration of nanomaterials, biomimetic receptors, Apts, and synthetic proteins are important characteristics. These sensors may be used with modern innovations such as lab-on-a-chip systems and provide enhanced selectivity, sensitivity, and reaction times. They are employed in many different industries for extremely sensitive detection [182].

Over time, glucose biosensors have become more dependable, quick, and precise. They have also become more portable and user-friendly. Research studies are still going on in the field of complicated technologies such as electrodes, membranes, immobilization techniques, and nanomaterials. Glucose monitoring is still hampered by a variety of issues, despite substantial advancements in glucose biosensor technology.

### 10.3 Point-of-care (POC) diagnostic devices

A POC diagnostic device is a tool used to collect particular clinical data about patients in healthcare settings with limited resources. The majority of users of POC diagnostic technologies are patients, their families, and medical professionals due to its ease of use. The advantage of POC is a drop in the turn-around time, or the amount of time that occurs between sample authentication and result publishing. The invention of a technique for quick blood glucose analysis in 1962 led to the introduction of POC testing [178]. Since then, POC testing has evolved and expanded to include a wide range of diagnostic tests for various diseases. These tests are particularly beneficial in resource-limited settings where access to laboratory facilities may be limited or non-existent. Additionally, POC testing allows for immediate diagnosis and treatment initiation, improving patient outcomes and reducing the burden on healthcare systems.

AuNPs coupled with a single-chain variable fragment antibody were developed by Rad and Colleges and were placed on a lateral flow immunoassay test strip. The study effectively illustrates the use of *Neisseria meningitidis* detection methods that are sensitive and quick. The bacterium that causes bacterial meningitis is *Neisseria meningitidis*. The test strip’s excellent sensitivity and quick reaction time are demonstrated by its ability to identify bacteria at a low concentration of 0.5 μg/mL in just over 3 min. The test strip is a potential technique for the early identification of bacterial meningitis because of its quick detection of low quantities of the bacterium. The promise of this diagnostic technique in clinical settings is reinforced by the validation of the structural integrity and binding effectiveness of the scFv antibody–AuNP complex using molecular docking and ProSA studies [183].

One of the main benefits of POC diagnostics is their low cost, as many Indians cannot afford the high cost of traditional tests. POC tests are highly useful for repeated follow-up testing to check treatment progress or for continuous monitoring of a specific analyte. For example, Oxford Biosensors (www.oxfordbiosensors.com) have created the Multisense® system, capable of detecting early signs of coronary heart disease. The fatality rate in the Euro-American world is high due to heart disease and is known to be correlated with a number of risk factors, including weight, diet, smoking, and cholesterol. A Multisense® system consists of a portable gadget with one-time-use electrochemical test strips. Numerous sensors on each strip independently detect both triglycerides and cholesterol (high-density and low-density lipoprotein), similar to a glucose sensor, monitoring blood glucose levels using a single drop of blood. Individuals at risk of heart disease can undergo quick and straightforward screening, diagnosis, and monitoring [184].

### 10.4 Environmental monitoring and bioremediation

According to UN population census estimates, India with population exceeding 1.4 billion, stands as the largest democracy globally [185]. Following its independence, the country has experienced remarkable progress, witnessing substantial enhancements in living standards. While still in the process of development, India has achieved noteworthy advancements in industrial growth, infrastructure, and its economy, positioning itself as a prominent global participant. However, these achievements have brought about environmental challenges. Environmental issues are major causes of illnesses, health issues, and long-term effects on livelihoods. Significant environmental issues that are currently affecting India are air pollution, poor waste management, growing water scarcity, falling groundwater levels, water pollution, and declining biodiversity. Various conventional methods are employed for the identification of heavy metals, phenolic compounds, pesticides, and other substances, such as gas chromatography (GC), high-performance liquid chromatography, and Fourier-transform infrared spectroscopy. The use of bioelectrochemical biosensors has increased due to their cost, labor-intensiveness, and restricted capacity to identify only one analyte. The first stage in cleaning the water and making it hygienic is to figure out the types and amounts of impurities in it (NCBI, 2021). It is not always clear that water will have distinguishable qualities such as taste, hue, or odor. Since polluted water lacks physical characteristics, it is thus important to analyze the physical and chemical parameters such as pH, conductivity, BOD, and COD of the water in order to quantify the amount of pollution and the pollutant load. In order to achieve these goals, an MFC can be designed as a BOD biosensor, which measures the biological oxygen demand of a water sample, and a COD biosensor, which measures the COD of a water treatment facility.

The use of air cathode single-chamber MFCs (SCMFCs) as a substitute measurement technique for BOD, a vital indicator of water quality. The lengthy BOD_5_ test is typically used to calculate BOD, which has drawbacks in terms of effectiveness and suitability for real-time monitoring. A high linear connection (*R* ^2^ > 0.99) was found between the total charge and the BOD_5_ content in experiments using simulated wastewater and sodium acetate as the fuel. Real household wastewater was used in the study to further validate this association, and the results showed a combined correlation with an *R* ^2^ value greater than 0.98. The results highlight SCMFC-based biosensors as a viable technique for real-time, on-site tracking of wastewater BOD contents. Compared to more conventional techniques, this strategy has benefits including ease of use, quick reaction times, and compatibility for real-time process control [186].

Xu et al. built constructed wetlands (CWs) integrated with Microbial Fuel Cells (CW-MFC) platform to enable on-site monitoring of COD throughout the water treatment process. This resulted in the creation of a unique COD biosensor based on MFC technology [187]. The MFC is built with an anode at the bottom to improve the oxidation of organic contaminants in the wastewater system and a cathode on top to extract oxygen from the surrounding air. According to the analysis, the COD concentration (acetate) and MFC output voltage demonstrated two linear connections. The output voltage varied from 101.99 ± 7.42 to 631.74 ± 7.41 mV for COD ranging from 0 to 500 mg L^−1^, and from 631.74 ± 7.41 to 668.46 ± 0.01 mV for COD ranging from 500 to 1,000 mg L^−1^ [187]. According to Kharkwal et al., a new kind of MFC-based BOD biosensor using MnO_2_ as the cathode was created in order to reduce oxygen. This biosensor demonstrated stability for 1.5 years, enabling continuous BOD monitoring, and is cost-effective. These models have the advantage of tracking BOD continuously for 1.5 years when the anodic chamber contains a modest quantity of oxygen [188]. Considering the unprecedented decline in environmental quality, it is critical to quickly identify dangerous substances through real-time, on site monitoring. These compounds have many harmful effects on people and other living things; thus, it is essential to evaluate them. Contaminated water supplies can expose people to heavy metals, which can cause kidney damage, neurological disorders, cancer, and other health problems. In order to protect human health, it is vital to put into action of effective wastewater treatment techniques (such as water deionization [189] and water filtration [190]) that can effectively remove or reduce heavy metal concentrations. To improve water filtration and removal, matrix membranes use metal-organic framework (MOF) architectures [191]. Because of their large surface area and adjustable characteristics, MOFs are the best options for eliminating pollutants from water. For instance, it has been demonstrated that heavy metal ions such as lead and cadmium may be successfully removed from water using a composite membrane composed of MIL-101(Cr) chromium terephthalate, an MOF and polyvinyl alcohol (synthetic biodegradable polymers, which have been described as super adsorbents for hazardous metals) [190]. Also, microorganisms have limited ability to eliminate or decrease heavy metals [192].

Liu and Cheng conducted research employing an SCMFC to examine the oxidized states of two metals, Fe^3+^ and Cr^6+^. The study revealed that a rise in the concentration of Cr^6+^ (10 mg/L) negatively affected the power generation capability of the MFC [193]. In recent years, electrochemical biosensors have developed a unique method employing Apts as bio-receptors to screen for heavy metals in biological samples. Mercury, or Hg, is the heavy metal that is most commonly found in environmental commodities and has received the greatest research attention among the major heavy metals. Recently, a specific aptasensor has been developed for the detection of mercury ions. The proposed aptasensor, featuring a detection limit of 0.0036 nM, identified Hg^2+^ across a concentration range from 0.01 to 5,000 nM. The researchers affirmed the viability of on-site identification of Hg^2+^ and proficiently identified mercury ions in water samples utilizing a self-assembled aptasensor [194].

When aquatic systems become more eutrophic, cyanobacteria-induced algal blooms produce toxic substances including microcystins and brevetoxins. These toxins should be detected as early as possible to support the life of other aquatic environments. In this context, an electrochemical aptasensor was employed to monitor the marine neurotoxic brevetoxin-2 sensitively. To improve the performance of the sensor, gold electrodes were used, and their surfaces were modified with a SAM of cysteamine [195]. The improvement of biosensors for monitoring environmental quality will involve the utilization of innovative nanocomposites, nanomaterials, and functionalization techniques. The demand for immediate tracking of pollutants will require the creation of novel sensing systems and their incorporation into aviation systems [196].

### 10.5 Food safety and quality control

For a contemporary food sector, food safety is a crucial concern. Different phases in the food production process can introduce contaminants, pathogens, poisons, and other substances into the food chain. For instance, they may be formed in food through chemical reactions with substances or gather there when food is being stored [197]. The food analysis takes place by using standard methods such as ultraviolet detection, mass spectrometry, chromatography, or fluorescence, either alone or in conjunction with other separation techniques, toward the end of food production [198]. There are several drawbacks to these conventional methods. First, tainted items may go through the whole manufacturing chain or even reach retailers before contamination is discovered since the analysis is done at the final stage of the process. Second, these techniques of analysis demand huge sample quantities, experienced staff, and are costly, time-consuming, and arduous [199]. Therefore, by offering quick on-site monitoring and real-time manufacturing process information, bioelectrochemical biosensors are employed to ameliorate this scenario. Since its detection technique and bio-interaction processes are well understood, bioelectrochemical biosensors have been employed extensively among other biosensors [200].

Algae and microorganisms are the main reason for food related health problems. As they produce toxins – bacterial, fungal, or algal-based – depending on their origin. Contamination by toxins can happen at any point in the food supply chain, during preservation, transportation, and manufacturing, and it is unavoidable and unpredictable [201]. For example, in a study by Qi et al., a label-free electrochemical aptasensor was developed for the identification of the small molecule saxitoxin (STX). STX is a primary toxin linked to paralytic shellfish poison, capable of causing shock, asphyxia, and even fatality within a lethal dose range. Utilizing the nanotetrahedron, the Apt-triplex used as bioreceptor, was immobilized onto the surface of a screen-printed electrode. The aptasensor exhibited robust practicality in authentic seawater samples, displaying well-confirmed recovery data (94.4–111%) and an LOD of 0.92 nM. It also showcased outstanding selectivity, stability, and repeatability [202].

Abnous et al. demonstrated an exceptional level of specificity, robustness, and reproducibility [203]. They developed an electrochemical aptasensor for identifying aflatoxin B1 (AFB1) in food items. The aptasensor functions by means of the interaction between Apt and complementary strands (CSs) of aptamer, which along with endonuclease I (Exo I) generate a π-shaped structure on the electrode surface. AFB1 is a carcinogenic secondary metabolite that primarily originates from Aspergillus flavus and Aspergillus parasiticus, and is classified as a Group 1 carcinogen by the International Agency for Research on Cancer and has the greatest toxicity of any aflatoxin family member. Under ideal circumstances, free ssDNA from its 3′-end is preferentially broken down by the sequence-independent enzyme exonuclease I (Exo I). With increasing AFB1 concentrations, the DPV signals were sharper, showing a dynamic range of 7–500 pg/mL and an exceptionally low LOD of 2 pg/mL [203].

Pathogens are microorganisms such as fungi, bacteria, and virus that are responsible for causing infectious diseases. They can enter the body through food, water, and air and are in charge of causing an increase in the world’s annual fatality rate [204]. In either way, whether pathogen is present in water or entering the human body can cause serious health conditions to humans. Since the conventional methods takes more time and are expensive, biosensors are employed for monitoring pathogens. An impedimetric immunosensor was created by Cimafonte et al. to detect *E. coli* in drinking water [205]. Warm-blooded animals and human’s natural digestive systems are home to *E. coli* bacteria that help the body to produce vital vitamins. On the other hand, some *E. coli* strains are capable of producing toxins, which can lead to negative outcomes such as anemia, stomachaches, and bloody diarrhea. Gold screen-printed electrodes (Au-SPEs) were used as the working, reference, and counter electrodes in a fluidic cell to build the immunosensor. Different solutions were added to the gold-plated area of the electrodes in order to measure the impedance. To activate anti-*E. coli* antibodies, the photochemical immobilization technique was used instead of SAM for antibody binding to gold surfaces. The gold surface of the Au-SPEs was treated with a solution containing UV-activated anti-*E. coli* antibodies. The signal was strengthened when anti-*E. coli* was delivered to the gold surface at a steady inflow rate, creating a sandwich complex. Utilizing both CV and EIS, a notable LOD of 3.0 × 10¹ CFU/mL was attained.

Nowadays clustered regularly interspaced short palindromic repeats (CRISPR), an endonuclease, a recently developed technology for genome modification, has many advantages in diagnostics. Moreover, CRISPR is employed to track SARS-CoV-2 through interactions between target genetic material and guide RNA that are specific to specific sequences [206]. When the endonuclease functions such as trans-cleaving were combined with bioelectrochemical output, an affordable system can be developed for monitoring the genetic material of pathogen. Liu and his co-workers developed a bioelectrochemical biosensor using CRISPR-Cas12a for the detection of human papillomavirus 16 and parvovirus B19 [122,207]. CRISPR-Cas12a, an RNA guided enzyme that is once guided to its target DNA, CRISPR-Cas12a can cut the DNA at that specific location without strict specificity, potentially affecting nearby DNA sequences as well. Hence, by modifying the guide RNA sequence enables the engineering of Cas12a to target DNA unique to a pathogen such as HPV [121,208]. Detecting the presence of HPV can be accomplished by using electrochemical methods to identify the endonuclease activity of Cas12a that has been triggered by the presence of HPV.

### 10.6 Industrial and bioprocess monitoring

A biosensor has wide application in the area of bioprocess industry. The bioprocess variables such as oxygen uptake rate, biomass concentration, cellular process, and so on happening in a bioreactor can be rapidly detected if a biosensor is implanted in the system. A bioprocess is a biochemical process in which a living system is provided with favorable conditions to produce products. These products can either enhance the function of existing organs, improve food products, or contribute to the production of therapeutic drugs. And the production will be in large scale process. So, continuous monitoring is essential to efficiently utilize time, chemicals, and biological reactors.

In the 1970s, bioprocess automation was introduced, specifically for the fermentation process involved in penicillin production. During that period, certain parameters such as pH, dissolved oxygen, and nutrient concentrations were not actively regulated in the fermentation culture [123,209]. One of the key elements of bioprocesses is biomass, which stands for solid samples or components measurements. Biomass can be described as overall mass of a living organism. That is biomass can be glucose, urea, lactate, and so on. Sun et al. developed a volatile fatty acid (VFA) monitoring bioelectrochemical sensor mainly for acetate detection during anaerobic digestion [210]. The novel microbial electrochemical biosensor is a three-chambered system; consisting of sample, anode, and cathode chambers separated by anion exchange membrane (AEM) and CEM, respectively. As the present acetate in the digestate travel from the sample chamber to the anodic chamber through AEM, electroactive bacteria oxidize the acetate, leading to the generation of electric current. VFAs, essential compounds in the pathways of fermentation and methanogenesis, function as dependable indicators of stress within the system. As a result, in the anaerobic digestion process, the buildup of VFAs may indicate an imbalance in the dynamics between producers and consumers [211]. The MFC-based biosensor developed in this study exhibited an improvement of around 20% in current densities at elevated temperatures (e.g., 37 and 55°C) after 5 h. As the external resistance decreased, the biosensor’s detection range expanded. Acetate concentrations measured by the biosensor were in close agreement, within 24.2%, with those determined by GC. This underscores the biosensor’s precision for real-time, on-site monitoring of VFAs in anaerobic digestion.

### 10.7 Monitoring biofuel production and fermentation processes

Bioelectrochemical biosensors are used in a wide range of sectors, such as biotechnology, food and beverage, healthcare, and environmental monitoring. By using the electrochemical reactions produced by biological processes, these biosensors play a crucial role in the identification and measurement of certain biological substances, or analytes. In healthcare, they find use in diagnostics and health parameter monitoring. Environmental monitoring employs these biosensors for detecting pollutants and contaminants. The food and beverage industry utilizes them for quality control and safety assessments. Moreover, bioelectrochemical biosensors contribute to research and development in the broader field of biotechnology.

The wine production industry plays a significant role in generating substantial economic revenue. Wine is an intricate blend of fruits (plum, strawberry, pomegranate), most commonly derived from grapes and various biochemical processes occurring throughout its production, spanning from alcoholic fermentation to the aging process [212]. Electrochemical biosensors have shown promise in the wine industry for tracking important parameters associated with malolactic fermentation and alcoholic fermentation. Evaluating the effects of different technological processes and remedies on wine performance, and identifying fluctuations resulting from wine age, varietal, and vineyard location would make it easier for winemakers to manage the manufacturing process and choose the wine’s ultimate flavor, quality, or taste. Voltammetry and amperometry are the most used electrochemical methods in analyzing wine quality [213]. Yeast is the main ingredient in the biochemical process to transform grape sugars into alcohol, carbon dioxide, and various metabolites for the production of wine, this process is known as alcoholic fermentation. Since this step is crucial in products’ final quality, many biosensors have to be developed to check the parameters such as glucose, glycerol, and fructose based on amperometric detection to meet the industry standards for robustness, precision, accuracy, etc. For example, the online general analyzer and the SIRE biosensors have been developed [214,215]. In the former, the enzyme is immobilized on membranes near the amperometric detector whereas in the latter, fresh enzyme was supplied in a liquid form within a compartment that was segregated from the sample by membranes. The process by which the lactic bacteria in wine convert l-malic acid to l-lactic acid is known as malolactic fermentation. This process results in a reduction in acidity of wine, which further shows up in the chemical and microbial stability of wines. Traditionally, wineries would measure the overall acidity or use a paper chromatograph to evaluate the lactic and malic acids semi-quantitatively. These can be easily detected by using enzyme amperometric biosensor. Gamella et al. reported a system for tracking the malolactic fermentation that included two enzymatic sensors for measuring lactic and malic acid. For construction they used enzymes, and tetrathiafulvalene (TTF), an electrochemical mediator [216]. Malic acid in the malolactic fermentation is converted to oxaloacetate by malate dehydrogenase and subsequently NAD^+^ is reduced to NADH, while another enzyme diaphorase reacts with NADH and mediator TTF, restoring NAD^+^. The reduced TTF is oxidized at the electrode surface and then polarized at a suitable potential. They noted that the current generated in the anode varies directly with the concentration of malic acid present in the sample. In the case of lactic acid determination, hydrogen peroxide undergoes conversion to water in the presence of TTF, facilitated by horseradish peroxidase. The resulting oxidized TTF is subsequently reduced on the electrode, polarized at −10.05 V. The resulting current in cathode is directly proportional to the lactic acid level in the sample.

### 10.8 Internet-of-things (IoT) integrated electrochemical biosensors

**Figure 6:**
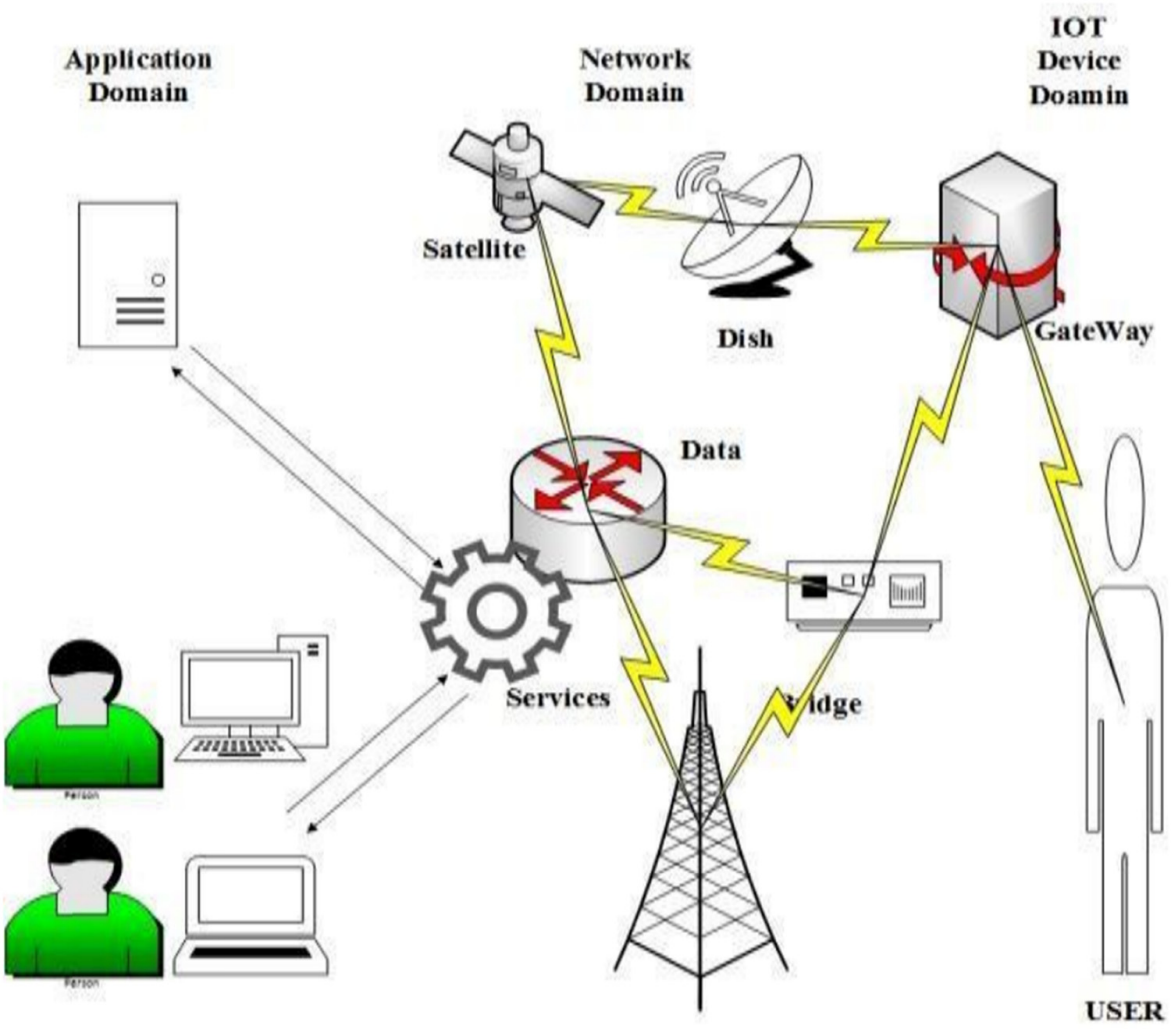
Illustration of the way in which the IoT links medical professionals, patients, and the wider network, guaranteeing that digital records are available to everybody [204].

An improvement in information technology that allows for process automation and human-free data transfer via wireless networks is called the IoT [217]. One aspect of IoT that is suggested to assist patients in receiving the right care is wearable technology. With the advent of 5G, a new set of technologies has emerged that provide the essential “backbone” for linking to the hundreds of millions of devices that will make up the impending IoT, which has the potential to drastically alter both our personal and professional lives [217]. The necessity for quick diagnostic technologies to identify and cure infectious illnesses has been brought to light by the worldwide pandemic. Devices known as POC testing, or POCT, are essential for promptly identifying infectious illnesses and presenting findings on the spot for treatment and early intervention. By providing real-time testing and quick, laboratory-caliber microbiological detection, they stop the spread of illness. An important development is the POCT’s connection with the Internet of Medical Things (IoMT), which enables wireless operation and connectivity with medical facilities and specialists as shown in Figure 6. The focus of recent advancements in POCT diagnostics and their integration with IoMT is on infectious illnesses that are developing or have resurfaced, such as COVID-19, HPV, dengue fever, malaria, Ebola, and Zika. POCT systems with IoMT support can close the knowledge gap between bioinformatics, fast analytics, and clinical validation, enhancing the understanding of illness development and treatment choices [218].

Innovative solutions are required to environmentally friendly urban water management due to the declining water quality. In order to enhance wastewater treatment and water quality, a study by Kumar and Hong, suggests a Surveillance-based sewage wastewater monitoring system (SSWMS) that uses IoT. Water quality, pressure, temperature, and other characteristics are tracked by the system using smart water sensors, which provide real-time data for tracking the movement of water throughout the treatment plant. The system guarantees that chemical emissions do not exceed allowable limits and evaluates the efficacy of the wastewater treatment process. According to experimental data, the SSWMS successfully lowers moisture content, improves recycling water quality to 97.98%, and provides secure communication. IoT integration in wastewater treatment enhances effective sewage disposal management, water quality assurance, and monitoring [219]. In place of manual techniques and conventional centralized systems, wireless sensor networks (WSNs) present a viable alternative for tracking water quality [220]. For deployment to be financially viable, a low-cost mobile sensor node system is required. In this work, a low-cost water quality measuring device that was tested in a wastewater treatment facility setting is integrated and validated within a WSN. The apparatus has a nitrate and nitrite analyzer that uses a cutting-edge ion chromatography detection technique. The gadget is put to test in real-world scenarios and incorporated utilizing an IoT programming platform. By enabling online connectivity with many devices throughout water treatment plants, an autonomous smart water quality surveillance system is established for efficient water control.

## 11 Performance evaluation and challenges

Examining operational parameters is essential, as it can reveal the nature of rate-limiting processes, be they related to transport or reaction. The characterization of a biosensor’s response holds equal importance in comprehending sensors relying on chemical recognition. Every biosensor exhibits specific static and dynamic attributes, and optimizing these properties directly influences the overall performance of the biosensor. In Sections 11.1–11.4, we will delve into critical performance requirements and elucidate their connections with the features of the receptor and transducer components in electrochemical biosensors.

### 11.1 Analytical performance parameters

In the world of emerging pollutants and growing population, nature and animals are only in the risk of unstable health conditions. Therefore, the importance of biosensors is evident. Analytical quality parameters are particular standards used to evaluate the dependability and efficiency of analytical tools and procedures. The accuracy, precision, and resilience of any analytical measurements are enhanced by these characteristics. Biosensors, regardless of their specific design or application, exhibit some common static and dynamic characteristics. These are discussed subsequently.

#### 11.1.1 Sensitivity, selectivity, and detection limits

In the context of a biosensor, sensitivity is defined as the lowest signal-to-noise ratio output that may be produced with a given minimum input signal amplitude. The measurement’s standard deviation determines this metric. Sensitivity is often determined by drawing a calibration curve from the measured current on the *Y*-axis and the concentrations of the particular analyte on the *X*-axis. The sensitivity of this curve is mostly determined by its slope. A higher slope value denotes a more sensitive reaction from the biosensor. To put it another way, a steeper slope highlights the biosensor’s capacity to pick up on minute fluctuations in the target analyte levels, indicating that minor changes in analyte concentration resulted in more noticeable variations in the measured current. There is always some confusion between LOD and sensitivity. LOD takes into account both the lowest analyte concentration, or blank, and the noise signal. In contrast, sensitivity refers to a biosensor’s reactivity.

#### 11.1.2 Selectivity

The essential component of a biosensor is its selectivity. It is the capacity of a bioreceptor to recognize a specific analyte in a sample that contains other contaminants and extraneous elements. In other words, the attraction of certain antigens toward a specific antibody. For example, if we consider glucose biosensor, it should specifically detect and interact with a particular analyte, i.e., glucose. In fruit juice, glucose has many competitors such as ascorbic acid, fructose, and citric acid which can interfere with the analyte [221]. The designed biosensor should breakdown glucose into hydrogen peroxide and gluconic acid. This shows the selectivity of GOx toward the glucose, not to any other substrate in juice. The selectivity of a biosensor is intricately linked to both the choice of the biological receptor and the selection of an appropriate transducer. Usually, the modification pertains to the sensitivity of the biosensor rather than its selectivity. There are two approaches for evaluating selectivity: one is by comparing the biosensor’s reaction to interfere substances with the analyte’s calibration curve, while the other entails introducing interference causing compounds straight in to the measuring cell. The latter method, while more straightforward to measure, is application-specific and can be influenced by the range of analyte concentrations [222].

#### 11.1.3 Detection limits

The detection limit, or lower LOD, is defined as the minimum amount of a substance discernible by the sensor output signal, distinct from the absence of that solute in the control. In simpler terms, it represents the smallest quantity ranging from 10^−^⁹ to 10^−^¹⁵ g of the material that the sensor can reliably identify, distinguishing it from a situation where the substance is entirely absent. For example, in a glucose biosensor, the lower limit of glucose concentration is that a biosensor can detect any background noise above the lower limit, which is 0.1 mmol/L. That means, below this limit, the biosensor cannot read the glucose concentration which is considered as the LOD of the glucose biosensor.

### 11.2 Response time and stability

#### 11.2.1 Stability

The stability of a biosensor refers to its susceptibility to environmental disturbances, which may induce a shift in the biosensor’s output signals during measurements [223]. In simpler terms, a biosensor should maintain consistent performance throughout its operational life. The ideal stability of a biosensor depends on factors such as the sensor’s shape, preparation technique, and the choice of receptor and transducer [222]. The substrate also influences biosensor stability. The substrate’s external environment, internal diffusion processes, or the nature of the biochemical sensing reaction will also have an effect on biosensor. For instance, in the case of a pH sensor used to measure acidity or alkalinity, a stable pH sensor will consistently provide accurate readings even after prolonged use. Calibration is the key for achieving high stability and accuracy, and it should be conducted regularly over time.

For applications requiring lengthy incubation phases or ongoing monitoring, stability is the most important characteristic of a biosensor. The bioreceptor’s gradual deterioration is another element that influences a measurement’s consistency. When evaluating the storage stability of a biosensor, key parameters include the storage condition (whether dry or wet), the atmospheric composition (whether air or nitrogen), pH levels, buffer composition, and the presence of additives [222].

#### 11.2.2 Response time

Response time of a biosensor is the time required by the sensor to produce an output signal with respect to the detected analyte. In biosensor, we consider both transient and steady-state response time. The latter is determined by the addition of each analyte in to the cell. It signifies the duration needed for the biosensor’s output signal to achieve 90% of its ultimate stable value following the introduction of an analyte [224]. Here the time interval shows how fast the biosensor comes back to its stable state after being disturbed by the action of analyte. The former one is defined as the maximum time required for the rate of change of output signal with respect to time, represented by (d*R*/d*t*) max, attain the highest value with respect to the addition of the analyte. Here the biosensor rapidly reflects in the presence of analyte and thus how quickly the signal starts to change.

When biosensors are utilized for consecutive measurements, whether in batch or continuous flow configuration, an essential metric is the sample throughput. This metric signifies the number of individual samples processed within a specified time frame. It takes into account not only the steady-state or transient response times but also includes the recovery time. It is the duration necessary for the signal to revert to its baseline after an event. Both response time types can be influenced by factors such as sample composition, analyte concentration, or the historical performance of the sensor. Systematically testing and quantifying these dependencies is crucial for gaining a comprehensive understanding of biosensor behavior [222].

#### 11.2.3 Biocompatibility and biofouling issues

The term biocompatibility with reference to biosensor defines about how well the sensor can interact with biological system without causing any harm. That is, the interaction of biosensor’s components with living tissue or fluids should not provoke immune response. For example, in glucose biosensor, the enzyme GOx should not induce any immune response when it comes in contact with the patient’s blood. Otherwise, it will affect the accuracy and precision of the biosensor. Hence, to avoid this, the material should be properly selected, enzyme should be compactible, implantation should be properly done, and make sure that biosensor does not release any harmful substance when in contact with biological interface. Ratner previously characterized biocompatibility as the material’s capability to elicit a subdued response, ensuring that the sensor is not entirely identified as a foreign entity [225].

For wearable biosensor used in healthcare, both mechanical and immune biocompatibility are important. Mechanical biocompatibility is used when a wearable sensor comes in contact with skin, that is at the time of its attachment. It is essentially based on lightweight, great flexibility, and exceptional stretchability. These features ensure steady and uninterrupted signal acquisition, permitting unhindered participation in dynamic bodily activities such as exercise. The wearable biosensors can be directly in contact with the skin or indirect. In such cases, it is crucial for wearable medical devices to pose no additional health risks and refrain from impeding daily activities [226]. This underscores the importance of ensuring the immune compatibility of wearable biosensors [227].

#### 11.2.4 Biofouling issues

Biofouling is the buildup of biological materials, including proteins, cells, or microorganisms, on the biosensor’s surface. Such accumulation can disrupt the sensor’s functionality and precision. Biofouling is a result of processes involving the non-specific or non-selective adsorption of various biological substances, such as proteins, cells, and oligonucleotides, as well as byproducts from neurotransmitter oxidation, and the precipitation/polymerization of phenolic compounds. Furthermore, these processes can have detrimental effects on the consistent electrochemical biosensing of important analytes and markers. These effects may occur either directly or following extended incubation in protein-rich samples or under extreme pH conditions [228]. Electrode is the main substance prone to biofouling in a bioelectrochemical biosensor. Fouling agents (amino acids, RNA, DNA) have a tendency to attach to the electrode surface via various interactions, including hydrophobic, hydrophilic, and electrostatic interactions. Soluble proteins are noteworthy fouling agents due to their dual nature: they exhibit hydrophilic behavior on the surface to interact with the watery environment and hydrophobic behavior internally to maintain protein structure. Consequently, soluble proteins have the capability to foul electrodes in a biosensor. These directly affect the analytical parameters of the biosensor [229]. Hence, to improve, antifouling strategies have been employed, such as utilizing a modified electrode with enhanced resistance and developing antifouling electrochemical biosensing interfaces. This involves the incorporation of materials such as polymers, hydrogels, peptides, and thiolated SAMs [230]. Alternative strategies to mitigate fouling encompass employing carbon materials, metallic NPs, catalytic redox couples, nanoporous electrodes, and electrochemical activation [228,231].

### 11.3 Scalability and cost-effectiveness considerations

Scalability of a biosensor refers to its ability to enhance performance across different scales, providing increased opportunities for diverse applications. In simple words, maximize the production of biosensor by maintaining its sensitivity and specificity, once the biosensor is effective in research laboratory, it can be further processed for large-scale industrial process. For example, in the case of a glucose biosensor, on a small scale, it can be employed to analyze glucose levels in blood samples. As the scale increases, such biosensors find applications in various industries such as pharmaceuticals, biofuels, and the wine industry, where they can be utilized to measure the glucose content specific to each industry. In environmental monitoring, these biosensors play a role in assessing glucose levels in water or soil. Additionally, in healthcare, they are essential for routine glucose monitoring in clinical settings.

### 11.4 Cost-effectiveness consideration

Cost-effectiveness is crucial in the development of biosensors, impacting their affordability and widespread availability. A cost-effective biosensor is one that can be manufactured at a reasonable cost without sacrificing its sensitivity and specificity. Several instances of cost-effective biosensors include a versatile algae-based biosensor designed for water quality analysis [232], fiber-optic biosensors enabling real-time detection in medical applications [233], and affinity-based electrical biosensors capable of sensitive and specific analyte detection in complex biofluids, all achieved at a low cost [234].

Fraser observed back in 1995 that the biosensor markets hold challenges that call for creative and innovative solutions [235]. Essentially, the future growth of biosensor markets hinges on finding a balance between market opportunities and overcoming technical or financial hurdles. According to a report by Kalorama Information (2006), four key factors could contribute to the expansion of the biosensor market: increased expectations for a better quality of life, the introduction of innovative technologies, ongoing research in industrial/academic Laboratories, and the gradual adoption of biosensor techniques to improve healthcare and diverse applications. Axela Biosensors, (Axela Biosensors, 2006), from Toronto, Ontario, Canada, specializes in commercializing products that aid in validating protein biomarkers, facilitating their transition from discovery to routine clinical use. Their DOT™ technology facilitates the instantaneous identification and measurement of protein binding events in intricate media. The DOT sensor, incorporating microfluidics and photonic technology, is both efficient and cost-effective.

Hence, cost-effectiveness of a biosensor depends on variety of factors such as materials and production costs, ease of fabrication and integration, integration with existing infrastructure, sensor longevity and reusability, scalability, waste management, and so on.

## 12 Conclusion

Bioelectrochemical biosensors offer a promising approach for real-time, *in situ* monitoring of wastewater. Biosensors play a vital role in ensuring timely and accurate detection of substances, ultimately contributing to the overall well-being of both humans and ecosystems. Additionally, biosensor technology has the potential to revolutionize various industries by providing real-time data, leading to more efficient and sustainable practices. In the medical field, the development of biosensor plays an important role in clinical diagnosis, where early detection is crucial for better treatment and diagnosis therapy. Another crucial area where biosensor technology is needed is environmental monitoring, for the quick detection of pesticidal residues, water quality maintenance, and air quality checks to avoid associated health risks.

Biosensors are a revolutionary development in wastewater monitoring, especially when it comes to measuring factors such as BOD and COD. Their unique combination of biological elements and state-of-the-art electrochemical technology provides unmatched benefits such as high sensitivity, quick response times, continual tracking, and continuous operating possibilities. The present review has encompassed an examination of diverse biosensors, based on bioreceptor and transducer part. Notable advancements in microbial community engineering, electrode material advancements, and optimization methodologies have been highlighted. The requirement for sensor resilience, reliability in a variety of climatic situations, and cost-effectiveness for scalable deployment means that despite these developments, obstacles still stand in the way of widespread use. In order to fully utilize biosensors to protect public health, advance environmental sustainability, and improve wastewater management techniques, it is essential to address these obstacles. Future generations will need cleaner and safer water supplies; therefore, biosensor technologies will need to be further refined and applied. This will require ongoing research and innovation.

Bioelectrochemical biosensors hold significant potential for revolutionizing industrial bioprocess monitoring. By addressing current challenges and exploring new avenues, these biosensors can contribute to more efficient, optimized, and cost-effective biomanufacturing processes across various industries.
